# Advanced Soft Porous Organic Crystal with Multiple Gas‐Induced Single‐Crystal‐to‐Single‐Crystal Transformations for Highly Selective Separation of Propylene and Propane

**DOI:** 10.1002/advs.202303057

**Published:** 2023-12-14

**Authors:** Lin Li, Shuhong Zhao, Huiming Huang, Muyao Dong, Jie Liang, Hui Li, Jian Hao, Engui Zhao, Xinggui Gu

**Affiliations:** ^1^ Beijing Advanced Innovation Center for Soft Matter Science and Engineering State Key Laboratory of Chemical Resource Engineering College of Materials Science and Engineering Analysis and Test Center Beijing University of Chemical Technology Beijing University of Chemical Technology Beijing 100029 P. R. China; ^2^ School of Science Harbin Institute of Technology Shenzhen HIT Campus of University Town Shenzhen 518055 P. R. China; ^3^ Beijing National Laboratory for Molecular Sciences Beijing 100190 P. R. China

**Keywords:** gas separation, gate opening, molecular‐scale shape‐memory effect, single‐crystal‐to‐single‐crystal, soft porous organic crystals

## Abstract

Soft porous organic crystals with stimuli‐responsive single‐crystal‐to‐single‐crystal (SCSC) transformations are important tools for unraveling their structural transformations at the molecular level, which is of crucial importance for the rapid development of stimuli‐responsive systems. Carefully balancing the crystallinity and flexibility of materials is the prerequisite to construct advanced organic crystals with SCSC, which remains challenging. Herein, a squaraine‐based soft porous organic crystal (SPOC‐SQ) with multiple gas‐induced SCSC transformations and temperature‐regulated gate‐opening adsorption of various C1‐C3 hydrocarbons is reported. SPOC‐SQ is featured with both crystallinity and flexibility, which enable pertaining the single crystallinity of the purely organic framework during accommodating gas molecules and directly unveiling gas‐framework interplays by SCXRD technique. Thanks to the excellent softness of SPOC‐SQ crystals, multiple metastable single crystals are obtained after gas removals, which demonstrates a molecular‐scale shape‐memory effect. Benefiting from the single crystallinity, the molecule‐level structural evolutions of the SPOC‐SQ crystal framework during gas departure are uncovered. With the unique temperature‐dependent gate‐opening structural transformations, SPOC‐SQ exhibits distinctly different absorption behaviors towards C_3_H_6_ and C_3_H_8_, and highly efficient and selective separation of C_3_H_6_/C_3_H_8_ (v/v, 50/50) is achieved at 273 K. Such advanced soft porous organic crystals are of both theoretical values and practical implications.

## Graphical abstract

By balancing the crystallinity and flexibility, multiple gases‐induced single‐crystal‐to‐single‐crystal (SCSC) transformations of a novel soft porous organic crystal (SPOC) are discovered for not only elucidation of gas‐framework interplays and molecular‐scale shape‐memory effect at molecular level but also highly selective separation of propylene/propane, demonstrating the crucial significance of stimuli‐responsive SCSC transformations in guiding development of stimuli‐responsive systems.

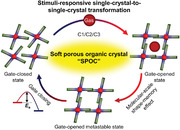

## 1 Introduction

Stimuli‐responsive structural transformations widely exist in nature, which inspired the development of advanced materials with adjustable structures and modulable functionalities under external stimuli.^[^ ^1^ ^]^ In particular, solid‐state phase transformations induced by chemical reactions or physical structure adjustments may lead to drastic changes in physical and chemical properties and have thus aroused tremendous interest in chemistry, material science, and engineering fields.^[^ ^2^ ^]^ Such structural transitions are usually highly dependent on their aggregation microenvironment and subtle variations of these microenvironments may exert profound influence on their responsiveness to specific stimuli or local environment.^[^ ^3^ ^]^ Single crystals with high structural regularity and great feasibility to be characterized by X‐ray diffraction (XRD) are ideal platforms to investigate these solid‐state phase transformations.^[^ ^4^ ^]^ However, these solid‐state structural transformations are mostly heterogeneous, which makes it difficult to provide insight into the structural transformation mechanism at the molecular/atomic level by single‐crystal X‐ray diffraction (SCXRD). In this sense, stimuli‐triggered single‐crystal‐to‐single‐crystal (SCSC) transformations without damaging the orders in the crystal lattice are of crucial implications,^[^ ^5^ ^]^ which enable revealing the structural evolutions by SCXRD technique and guide the design of solid‐state stimuli‐responsive systems with emerging properties for high‐tech applications.^[^ ^6^ ^]^ For SCSC systems, structural flexibility is of paramount importance for retaining the single crystallinity during structural transformations, which, however, apparently conflicts with crystal regularity.^[^ ^7^ ^]^ Thus, balancing the structural flexibility and crystal lattice regularity is the key to developing stimuli‐responsive SCSC transformations.

Flexible frameworks, of which structural transformations occur in a concerted manner, have attracted considerable attentions in guest‐induced structural transitions and were extensively applied in molecular recognition and separation technologies.^[^ ^8^ ^]^ Flexible metal‐organic frameworks (FMOFs) were reported to demonstrate desirable guest‐induced SCSC transformations, such as “gate‐opening” and “breathing,”^[^ ^9^ ^]^ in which the structural transformations were achieved by the configuration adjustment of organic ligands.^[^ ^10^ ^]^ Besides, some organometallic complexes with unique metal–metal interactions also exhibited reversible guest‐induced SCSC transformations, especially vaporchromic SCSC transitions.^[^ ^11^ ^]^ As FMOFs and organometallic complexes are constructed by coordination bonds and metal–metal interactions, achieving SCSC is practicable but the degree of transformation is also limited. In contrast, frameworks constructed by purely organic molecules are held together by non‐covalent interactions with significantly weaker bond strength than covalent and coordination bonds, such as hydrogen bonds, π–π interactions, and C─H···π interactions, exhibiting lower activation energy and more flexible possibility.^[^ ^7b^ ^]^ Thus, they are conducive to undergoing a large degree of transformation under external stimuli.^[^ ^12^ ^]^ More meaningfully, purely organic framework crystals with stimuli‐responsive SCSC transformations might adapt different guests with different sizes and functional groups, which would greatly promote the development of both distinctive host–guest chemistry and emerging functional materials.^[^ ^13^ ^]^ However, most purely organic crystals are fragile and easily collapsed, and tend to lose single crystallinity during structural transformation. Despite the extensive endeavors devoted,^[^ ^14^ ^]^ it remains challenging to concert the changes of various weak and flexible noncovalent interactions, and successful examples of purely organic SCSC have rarely been reported. This seriously impedes the understanding of host‐guest interplays and structural transformation mechanisms, which is crucial to the development of advanced stimuli‐responsive materials with SCSC transformations.

Recently, our group reported a novel soft porous organic crystal (SPOC) based on squaraine (SPOC‐SQ), which demonstrated fascinating guest‐induced SCSC transformation.^[^ ^15^ ^]^ We were motivated to explore the potential of such unique structural transformation for host–guest chemistry and high‐tech applications. Herein, we report the stimuli‐dependent SCSC transformations of SPOC‐SQ induced by multiple hydrocarbons of C1–C3 components with different molecular structures such as CO_2_, C_2_H_2_, C_2_H_4_, C_2_H_6_, C_3_H_4_, and C_3_H_6_, accompanied with temperature‐regulated gate‐opening sorptions. During these transformations, gas molecules were orderly accommodated in the purely organic framework, and single crystallinity was retained throughout the transformation, which enabled directly unveiling the gas‐framework interplays by SCXRD technique. More interestingly, multiple metastable single crystals after gas release were obtained, revealing the unique molecular‐scale shape‐memory effect (MSME) of SPOC‐SQ framework. By virtue of its single crystallinity, SCXRD unveiled the molecule‐level structural evolution of SPOC‐SQ crystal framework after gas departure and demonstrated its unprecedented flexibility and adjustability in conventional organic framework crystals. Thanks to the temperature‐dependent gate‐opening structural transformation, highly efficient and selective separation of C_3_H_6_/C_3_H_8_ (v/v, 50/50) was achieved at 273 K.

## 2 Results and Discussion

**Figure 1:**
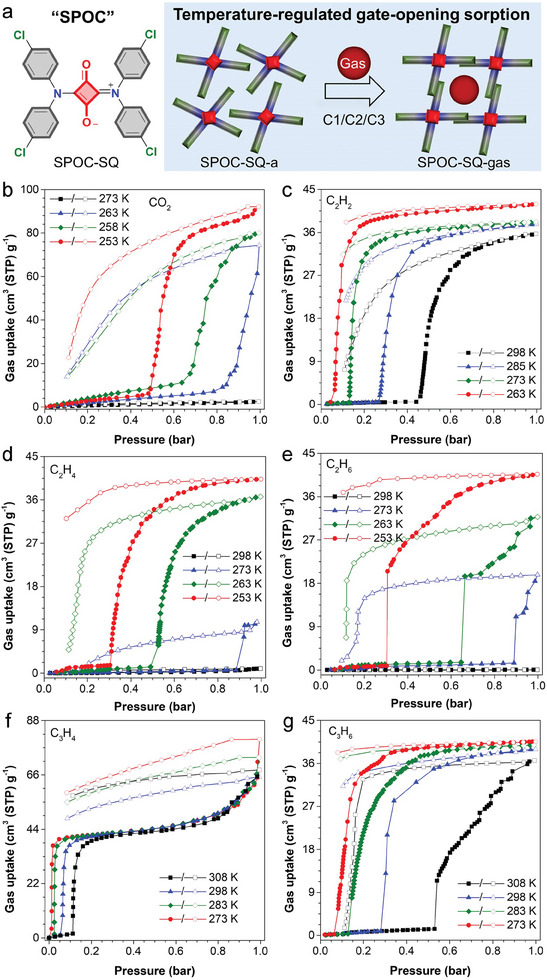
a) The chemical structure of SPOC‐SQ and the schematic illustration of temperature‐dependent gate‐opening sorption of C1, C2, and C3 hydrocarbons. Sorption isotherms of SPOC‐SQ‐a for b) CO_2_, c) C_2_H_2_, d) C_2_H_4_, e) C_2_H_6_, f) C_3_H_4_, and g) C_3_H_6_ at different temperatures. Filled and open symbols represent adsorption and desorption, respectively.

As gas adsorption ability was correlated to the structural flexibility of porous materials,^[^ ^7b^ ^]^ the gas sorption behavior of SPOC‐SQ‐a single crystals was first investigated. The sorption experiments of SPOC‐SQ‐a were carried out on light hydrocarbons of C1 (CO_2_ and CH_4_), C2 (C_2_H_2_, C_2_H_4_, and C_2_H_6_) and C3 (C_3_H_4_, C_3_H_6_, and C_3_H_8_) (**Figure** **1**). Except for CH_4_ with almost no gate‐opening sorption (Figure S1, Supporting Information), SPOC‐SQ‐a exhibited abrupt adsorptions of CO_2_, C_2_H_2_, C_2_H_4_, C_2_H_6_, C_3_H_4_, and C_3_H_6_ over certain pressures, presenting temperature‐dependent gate‐opening sorption characteristics (Figure 1a). These sorption isotherms were featured with typical hysteresis and the gate‐opening pressures were gas‐ and temperature‐dependent. Generally speaking, low temperatures were favorable for gas adsorption, suggesting the exothermic nature of gas adsorption processes. The adsorption of CO_2_ by SPOC‐SQ‐a crystals was induced at temperatures of below 273 K (Figure 1b), while the gate‐opening pressure for C_2_H_2_ was about 0.12 bar at 273 K and it could still be gate‐opened under 0.48 bar at 298 K (Figure 1c). Other C2 hydrocarbons (C_2_H_4_ and C_2_H_6_) required higher gate‐opening pressures of 0.88 and 0.89 bar at 273 K, respectively, and no obvious adsorption was detected at 298 K under pressures of up to 1 bar (Figure 1d,e), clearly demonstrating the more difficult gate‐opening adsorption of C_2_H_4_ and C_2_H_6_ with nonlinear molecular structures than that of C_2_H_2_ with linear molecular structure. For C3 hydrocarbons, the gate‐opening pressures were nearly 0 bar at 273 K for both C_3_H_4_ and C_3_H_6_ (Figure 1f,g). At a high temperature of 308 K, SPOC‐SQ‐a crystals could still adsorb C_3_H_4_ and C_3_H_6_ with low gate‐opening pressures of about 0.1 bar and 0.55 bar, respectively, revealing their high affinity towards SPOC‐SQ‐a for easy gate‐opening sorption. In contrast, C_3_H_8_ was difficult to be adsorbed by SPOC‐SQ‐a even at 253 K (Figure S2, Supporting Information), presumably due to its large steric hindrance. These results demonstrated the stimuli‐dependent gate‐opening responses of SPOC‐SQ‐a towards gases with different molecular structures.

Gate‐opening enthalpy (∆*H*_GO_) refers to the isosteric heat of adsorption upon gate‐opening.^[^ ^7c^ ^]^ It could be regarded as an important indicator to unveil the interplays between guest and host, and provide insights into the gas‐framework interactions, thus significantly facilitating us to understand the distinct gate‐opening sorption behaviors. To understand the stimuli‐dependent gate‐opening processes of SPOC‐SQ‐a, ∆*H*_GO_ values for different gases were determined. By plotting the gate‐opening pressure against temperature, the ∆*H*_GO_ values of SPOC‐SQ‐a for CO_2_, C_2_H_4_, C_2_H_6_, C_3_H_4_, and C_3_H_6_ were obtained by using the well‐known Clausius‐Clapeyron equation (Figure S3, Supporting Information).^[^ ^13c^ ^]^ Negative ∆*H*_GO_ values were obtained for all gases, which was consistent with the exothermic adsorption of SPOC‐SQ‐a crystals. The ∆*H*_GO_ values for CO_2_, C_2_H_2_, C_2_H_4_, and C_2_H_6_ were −30.62 kJ mol^−1^, −33.0 kJ mol^−1^,^[^ ^15^ ^]^ −30.66 kJ mol^−1^, and −30.98 kJ mol^−1^, respectively, which were well consistent with the abovementioned phenomenon that the gate‐opening process for C_2_H_2_ was easier than CO_2_, C_2_H_4_, and C_2_H_6_. Larger ∆*H*_GO_ values of −43.66 kJ mol^−1^ and −41.85 kJ mol^−1^ were obtained for C_3_H_4_ and C_3_H_6_, respectively. As a higher ∆*H*_GO_ could more readily overcome the energy requirement for phase transition at lower pressures, the larger ∆*H*_GO_ values of C_3_H_4_ and C_3_H_6_ were in line with the fact that C_3_H_4_ and C_3_H_6_ were more prone to undergo gate‐opening sorption.

**Figure 2:**
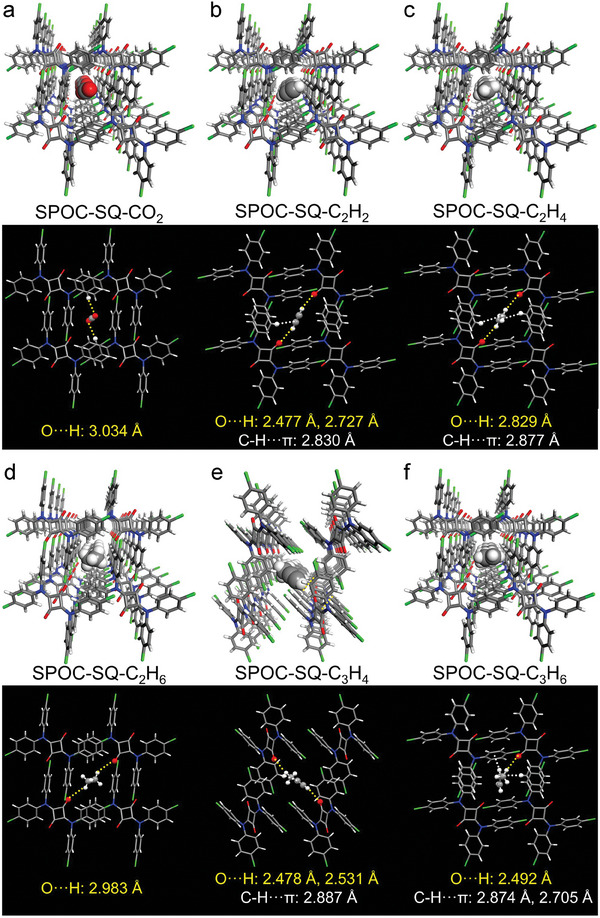
The packing diagrams and gas‐framework interactions of SPOC‐SQ single crystals including a) CO_2_, b) C_2_H_2_, c) C_2_H_4_, d) C_2_H_6_, e) C_3_H_4_, and f) C_3_H_6_. The structures and packing are determined by SCXRD. Gray, blue, red, green, and white represent C, N, O, Cl, and H, respectively.

According to the abovementioned gate‐opening sorption isothermals, SPOC‐SQ‐a single crystals could be gate‐opened to adsorb the gases of CO_2_, C_2_H_2_, C_2_H_4_, C_2_H_6_, C_3_H_4_, and C_3_H_6_ under different adsorption temperatures at 1 bar (Figure S4 and Table S1, Supporting Information), resulting in gas‐accommodated SPOC‐SQ‐gas single crystals, named as SPOC‐SQ‐CO_2_, SPOC‐SQ‐C_2_H_2_, SPOC‐SQ‐C_2_H_4_, SPOC‐SQ‐C_2_H_6_, SPOC‐SQ‐C_3_H_4_, and SPOC‐SQ‐C_3_H_6_ single crystals, respectively. Thanks to the great flexibility of the SPOC‐SQ framework, all these SPOC‐SQ‐gas maintained their crystal singularity during gate‐opening structural transformations (crystallographic data were summarized in Tables S2–S7, Supporting Information), which was essential for SCXRD analysis and could provide precise structural information regarding the gate‐opening process. All these crystals were in the same triclinic *P*‐1 space group. Crystallographic results indicated that SPOC‐SQ adjusted molecular conformation to form porous crystal frameworks through π–π and hydrogen bonding interactions with orderly arranged gas inclusions (**Figure** **2** and Figures S5–S9, Supporting Information), which was typical SCSC transformation. The gas molecules were trapped orderly in the cavity between two layers along 1D irregular pore channels through hydrogen‐bonding and C─H···π interactions. Due to the weak non‐covalent interactions in this purely organic system, SPOC exhibited great flexibility to adapt gas molecules with different shapes and sizes (Table S8, Supporting Information), which led to the gradually expanded cell volumes of SPOC‐SQ‐gas single crystals with the accommodated gases. Among them, SPOC‐SQ‐C_2_H_6_ and SPOC‐SQ‐C_3_H_6_ single crystals exhibited large cell volumes of 681.5 Å^3^ (Table S5, Supporting Information) and 690.4 Å^3^ (Table S7, Supporting Information), respectively, due to their bulky molecular structures. Interestingly, accommodating C_3_H_4_ into SPOC produced SPOC‐SQ framework with different channel shape from other gases, which should be ascribed to the long and rigid molecular structure of C_3_H_4_ (Figure 2e and Figure S8, Supporting Information). These results clearly demonstrated the great flexibility of SPOC‐SQ, which enabled it to function as an adaptive single‐crystal structure for accommodating gases with different sizes and molecular structures.

Benefiting from the single crystalline state of these SPOC‐SQ‐gas crystals, the host‐guest interactions could be facilely analyzed to provide insight into their different gate‐opening processes. It was found that the hydrogen bonds between the hydrogen of gas molecules and the electronegative oxygen of SPOC‐SQ played the dominant role in stabilizing gas molecules inside the SPOC‐SQ framework. In the SPOC‐SQ‐CO_2_ single crystal, the shortest distance between CO_2_ molecule and SPOC framework was 3.034 Å (C─O···H) (Figure 2a). Such long‐range interaction was extremely weak for efficiently accommodating CO_2_, which resulted in its relatively difficult gate‐opening sorption with small ∆*H*_GO_. For C_2_H_2_, C_2_H_4_, and C_2_H_6_, the gas‐framework interactions were obviously decreased, and the hydrogen‐bonding interactions became weaker gradually, as the Lewis acidity of C─H terminal bonds was decreased from C_2_H_2_, C_2_H_4_, to C_2_H_6_ (Figure 2b–d, SPOC‐SQ‐C_2_H_2_: 2.727 Å and 2.477 Å, SPOC‐SQ‐C_2_H_4_: 2.829 Å, and SPOC‐SQ‐C_2_H_6_: 2.983 Å). The hydrogen bonds between C3 gases and framework were even stronger (2.478 Å and 2.531 Å in SPOC‐SQ‐C_3_H_4_ and 2.492 Å in SPOC‐SQ‐C_3_H_6_) (Figure 2e,f), which could facilitate their gate‐opening sorption. Besides hydrogen bonds, some weak C─H···π interactions also existed in these SPOC‐SQ‐gas single crystals (2.830 Å for SPOC‐SQ‐C_2_H_2_, 2.877 Å for SPOC‐SQ‐C_2_H_4_, 2.887 Å for SPOC‐SQ‐C_3_H_4_, 2.874 Å and 2.705 Å for SPOC‐SQ‐C_3_H_6_), which could further immobilize gas molecules inside frameworks. These non‐covalent gas‐framework interactions in SPOC‐SQ‐gas single crystals not only benefited gas molecules accommodation for SCXRD analysis, but also contributed to the gate‐opening processes. The stronger gas‐framework interactions would induce larger ∆*H*_GO_ for easier phase transition, resulting in the lower gate‐opening pressure.

**Figure 3:**
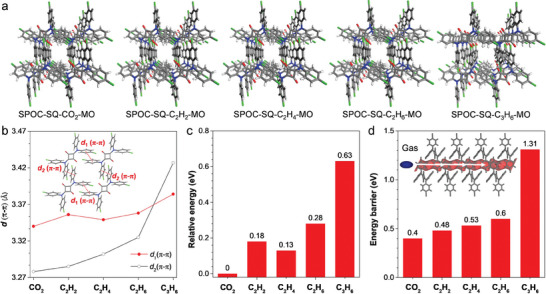
a) The intermediate gate‐opened framework SPOC‐SQ‐MO single crystals after gas removal. b) Plots of the π–π interactions between SPOC‐SQ molecules. Inset: illustration of the crucial π–π interactions to maintain the intermediate gate‐opened SPOC‐SQ‐MO framework. c) Relative energies of the unit cells in the intermediate gate‐opened frameworks by DFT calculations within SPOC‐SQ‐gas‐MO single crystals. For C_3_H_6_, the average distances of π–π interactions depicted in Figure S11 (Supporting Information) were used in this plot. d) Energy barriers against different gases that go through the 1D channel of SPOC‐SQ‐gas framework. MO: metastable open phase. Gray, blue, red, green, and white represent C, N, O, Cl, and H, respectively.

Subsequently, the framework structure evolution of SPOC upon gas removal was investigated (**Figure** **3**). After CO_2_, C_2_H_2_, C_2_H_4_, C_2_H_6_, and C_3_H_6_ gases were released, these SPOC‐SQs remained to be in single crystalline state without obviously collapsing and were immediately subjected to SCXRD analysis (SPOC‐SQ‐gas‐MO: SPOC‐SQ‐CO_2_‐MO, SPOC‐SQ‐C_2_H_2_‐MO, SPOC‐SQ‐C_2_H_4_‐MO, SPOC‐SQ‐C_2_H_6_‐MO, and SPOC‐SQ‐C_3_H_6_‐MO, Figure 3a and Tables S2–S5 and S7, Supporting Information). Surprisingly, after removal of gases their frameworks did not go back to their original state immediately and the channels were retained with decreased cell volumes, while for SPOC‐SQ‐C_3_H_4_‐MO, we tried several times to obtain its single crystal but failed as it quickly transformed to SPOC‐SQ‐a. These metastable gate‐opened frameworks upon gas removal were still in the triclinic *P*‐1 space group and were also constructed through π–π interactions and multiple hydrogen bonds (Figures S10 and S11, Supporting Information). Though gas molecules were removed, their molecular shapes and structures would affect the SPOC‐SQ‐gas‐MO crystal frameworks, resulting in different extents of π–π interactions between SPOC‐SQ molecules. As the molecular sizes of gases were increased gradually, the resulting SPOC‐SQ‐gas‐MO demonstrated weaker π–π interactions to maintain the intermediate gate‐opened framework for linear gas molecules (CO_2_ and C_2_H_2_) and nonlinear ones (C_2_H_4_, C_2_H_6_, and C_3_H_6_) (Figure 3b). As a result, their energies were gradually increased with decreased stabilities (Figure 3c). Removal of C_3_H_6_ molecules with both planar double bonds and bulky methyl groups called for the greatest adjustment of framework compared to planar C_2_H_4_ and bulky C_2_H_6_, and produced the most unstable intermediate gate‐opened framework with the highest energy (Figure 3c). These results suggested that the different stabilities of SPOC‐SQ‐gas‐MO single crystals should be attributed to the different disturbance of gases to frameworks during gas removal. To further verify the disturbance of gas to the framework, we employed DFT calculation to investigate the energy barriers upon gas passing through the channel of framework. For nonlinear C_2_H_4_, C_2_H_6_, and C_3_H_6_, the energy barriers were 0.53, 0.60, and 1.31 eV, respectively, while those for linear CO_2_ and C_2_H_2_ were 0.40 eV and 0.48 eV,^[^ ^15^ ^]^ respectively (Figure 3d and Figure S12 and Table S9, Supporting Information), which obviously demonstrated the larger disturbance of the nonlinear molecules to the framework than that of the linear molecules.

Afterwards, we investigated the stability of these SPOC‐SQ‐gas‐MO single crystals. Remarkably, the characteristic diffraction patterns of SPOC‐SQ‐CO_2_‐MO and SPOC‐SQ‐C_2_H_2_‐MO microcrystalline after CO_2_ and C_2_H_2_ removal in *in*‐*situ* powder X‐ray diffraction (PXRD) were unchanged for 5 h at room temperature and even after vacuum treatment, while the SPOC‐SQ‐gas‐MO single crystals for C_2_H_4_, C_2_H_6_ and C_3_H_6_ were not thermodynamically stable at room temperature, and could spontaneously transform to the original SPOC‐SQ‐a (Figure S13, Supporting Information). The result was suggestive of the great stability of these single crystals during treatment. We also conducted DSC analysis to study the gate‐closing processes, and broad endothermic peaks were obviously observed above room temperature. The activation energies (*E*_a_) were evaluated to be 42.04 kJ mol^−1^ and 38.81 kJ mol^−1^ for SPOC‐SQ‐CO_2_‐MO and SPOC‐SQ‐C_2_H_2_‐MO, respectively (Figure S14, Supporting Information), while their energies were larger than the activated SPOC‐SQ‐a single crystal according to the results of DFT calculations, demonstrative of their dynamically stability at room temperature. Besides, in five consecutive sorption cycles of these three gases, the sorption curves were typical gate‐opening isotherms with large hysteresis loops in the first cycle, and were converted to type I during the subsequent cycles (Figure S15, Supporting Information), revealing the unique molecular‐scale shape‐memory effect (MSME).^[^ ^16^ ^]^ This should be attributed to the larger disturbance of C_2_H_4_, C_2_H_6_, and C_3_H_6_ to the framework than that of CO_2_ and C_2_H_2_, which triggered easier and faster structural deformation to SPOC‐SQ‐a after gas releasing. Especially, the framework structure of SPOC‐SQ‐C_3_H_6_‐MO was slightly different from other SPOC‐SQ‐gas‐MOs, which could be ascribed to its large molecular structure and strong interactions with framework, facilitating the fastest structural transformation after C_3_H_6_ removal. Despite of the different effect of CO_2_, C_2_H_2_, C_2_H_4_, C_2_H_6_, and C_3_H_6_ on the frameworks, all their structural evolution processes underwent SCSC transformations, which were fundamental for the above molecule‐level understanding.

**Figure 4:**
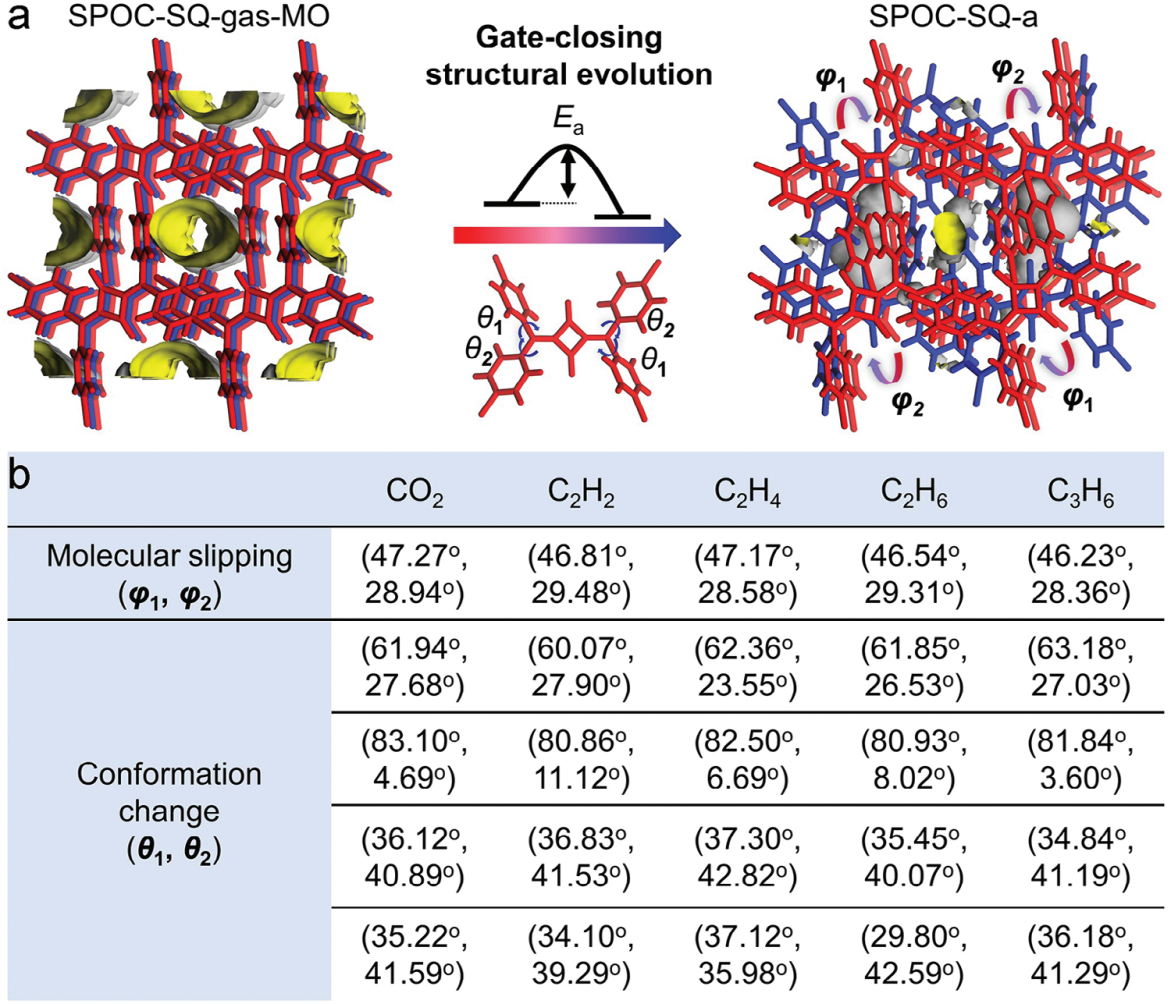
a) Illustration of gate‐closing structural evolution for SPOC‐SQ single crystals after gas removal (SPOC‐SQ‐gas‐MO) to gate‐closed activated state SPOC‐SQ‐a. b) Summarization of the slipping of the whole SPOC‐SQ molecules around the center of four‐member ring (*φ*) and rotation of terminal phenyl rings (*θ*) during gate‐closing structural evolution. All data were acquired by SCXRD. MO and a represent metastable open and activated closed phases, respectively.

To unveil the structural evolution of the gate‐closing processes, we compared the single crystal structures of SPOC‐SQ‐gas‐MOs and SPOC‐SQ‐a and discovered their complicated structural transformations with synergetic molecular motions. Among them, two dominant molecular motions of slipping for the whole SPOC‐SQ molecule around the center of the four‐member ring (*φ*) and rotation of terminal phenyl rings (*θ*) were crucial to the gate‐closing process (**Figure** **4**a). During the gate‐closing structural evolution, all SPOC‐SQ‐gas‐MO frameworks could transform to the stable SPOC‐SQ‐a state through two kinds of molecular slipping and four types of molecular conformation changes with different *φ* and *θ* values (Figure 4b and Figures S16–S25, Supporting Information), accompanied with gate closing of the one‐dimension channel. Remarkably, the intermediate short‐lived semi‐open single crystal phases (SPOC‐SQ‐gas‐MSO) were captured for C_2_H_4_ and C_2_H_6_ (SPOC‐SQ‐C_2_H_4_‐MSO (Table S4, Supporting Information) and SPOC‐SQ‐C_2_H_6_‐MSO (Table S5, Supporting Information)) between SPOC‐SQ‐gas‐MO and SPOC‐SQ‐a (Figures S26–S29, Supporting Information). These data clearly demonstrated the structural transformations during gate‐closing structural evolution at the molecular level, which has rarely been reported in purely organic crystals.

**Figure 5:**
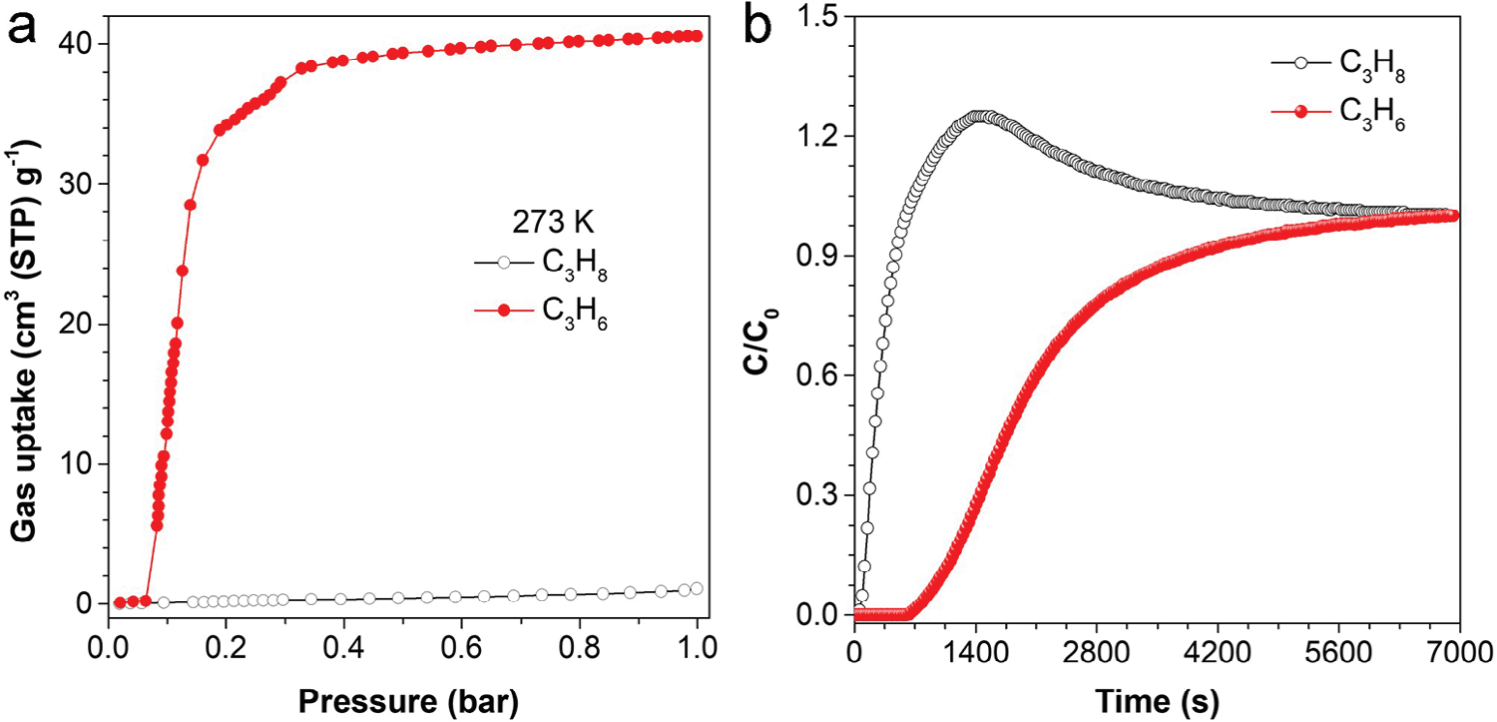
a) Sorption isotherms of SPOC‐SQ‐a for C_3_H_6_ and C_3_H_8_ at 273 K. b) Experimental column breakthrough curves for C_3_H_6_/C_3_H_8_ (v/v, 50/50) separation on SPOC‐SQ‐a (4.21 g) at 273 K and 1 bar in a column (inside diameter, 10 mm) at a flow rate of 5 cm^3^ min^−1^.

The unique structural flexibility and the induced‐fit aggregation microporous environment of SPOC‐SQ‐a prompted us to evaluate its potential for gas separation. Thanks to the distinct gate‐opening processes of SPOC‐SQ at different temperatures for different gases, their gate‐opening pressure could be regulated by temperature and efficient separation of multicomponent mixture could be achieved. According to their single‐component adsorption isotherms, the gate‐opening adsorption of SPOC‐SQ‐a crystals for C_3_H_6_ could be triggered under 0.06 bar at 273 K, exhibiting significant C_3_H_6_ uptake capacities (≈ 40 cm^3^ g^−1^). Under the same conditions, almost no C_3_H_8_ was absorbed (**Figure** **5**a), which enabled the effective separation of C_3_H_6_ from C_3_H_8_. Then, the separation efficiency of SPOC‐SQ‐a crystals towards C_3_H_6_/C_3_H_8_ mixture was further evaluated by the Henry selectivity at 273 K, and a Henry selectivity of 51.16 was obtained for C_3_H_6_/C_3_H_8_ at 273 K (Figure S30 and Table S10, Supporting Information), which demonstrated its remarkable selectivity at the corresponding temperature. This high selectivity of SPOC‐SQ‐a should be originated from the strong gas‐framework interactions of C_3_H_6_ as compared to that of C_3_H_8_ at such temperature. Then the breakthrough experiments were carried out in a dynamic binary gas mixture of C_3_H_6_/C_3_H_8_ (v/v, 50/50) at 273 K (Figure 5b). C_3_H_8_ was eluted at the very beginning, indicating neglectable adsorption of C_3_H_8_ in the column, whereas a long retention time of about 7000 s was observed for C_3_H_6_, demonstrating the good separation performance of SPOC‐SQ‐a crystals, making SPOC‐SQ excellent candidate for industrial separation of C_3_H_6_/C_3_H_8_ in low‐cost and energy‐efficient fashion and overcoming the intrinsically difficulties due to the similar physical and chemical properties between C_3_H_6_ and C_3_H_8_.

## 3 Conclusion

To sum up, multiple gases‐induced and temperature‐regulated SCSC transformations towards various C1–C3 hydrocarbons (CO_2_, C_2_H_2_, C_2_H_4_, C_2_H_6_, C_3_H_4_, and C_3_H_6_) were achieved in SPOC‐SQ crystals, accompanied with temperature‐dependent gate‐opening sorption. During these SCSC transformations, six C1–C3 hydrocarbons with different sizes ranging from CO_2_ to C_3_H_6_ could be successfully accommodated into the highly flexible SPOC‐SQ single crystal with retained crystal singularity, and the gas‐framework interplays could be directly revolved by SCXRD technique for molecule‐level elucidation of these unique gate‐opening SCSC transformations and gas‐absorption behaviors. Fortunately, after gas departure multiple metastable gate‐opened single crystals were successfully captured during the structural evolution, which enabled the detailed investigation on the MSME processes and the molecule‐level understanding of structural evolution for SPOC‐SQ crystal framework after gas departure. To our best knowledge, this is for the first time to achieve in purely organic porous single crystals constructed by π–π and hydrogen‐binding interactions. Finally, high‐efficiency and selective separation of C_3_H_6_/C_3_H_8_ (v/v, 50/50) was achieved at 273 K based on this temperature‐dependent gate‐opening structural transformation, which would be of significant implications in industrial applications. Collectively, this finding in this work is expected to deepen our understanding of the solid‐state stimuli‐responsive systems and promote the development of novel stimuli‐responsive materials.

[CCDC 2220252–2220269 contain the supplementary crystallographic data for this paper. These data can be obtained free of charge from The Cambridge Crystallographic Data Centre via www.ccdc.cam.ac.uk/data_request/cif.]

## Conflict of Interest

The authors declare no conflict of interest.

## Supporting information

Supporting Information

## Data Availability

The data that support the findings of this study are available from the corresponding author upon reasonable request.
