# Supplementary material for: Advanced Soft Porous Organic Crystal with Multiple Gas‐Induced Single‐Crystal‐to‐Single‐Crystal Transformations for Highly Selective Separation of Propylene and Propane
Source: Adv Sci (Weinh). 2023 Dec 14;11(9):2303057. doi: 10.1002/advs.202303057 (PMC10916656; doi:10.1002/advs.202303057)
Supplement: Supplementary file 1 — Supporting Information [file ADVS-11-2303057-s001.pdf]

ADVANCED SCIENCE
Open Access

# Supporting Information

for *Adv. Sci.*, DOI 10.1002/advs.202303057

Advanced Soft Porous Organic Crystal with Multiple Gas-Induced Single-Crystal-to-Single-Crystal Transformations for Highly Selective Separation of Propylene and Propane

*Lin Li, Shuhong Zhao, Huiming Huang, Muyao Dong, Jie Liang, Hui Li, Jian Hao, Engui Zhao* and Xinggui Gu**

Supporting Information

**Advanced Soft Porous Organic Crystal with Multiple Gas-Induced Single-Crystal-to-Single-Crystal Transformations for Highly Selective Separation of Propylene and Propane**

*Lin Li, Shuhong Zhao, Huiming Huang, Muyao Dong, Jie Liang, Hui Li, Jian Hao, Engui Zhao*, and Xinggui Gu**

**Experimental procedures**

**Materials and instruments**

Single-crystal X-ray diffraction (SCXRD) data were collected on a Gemini E X-ray diffractometer (Agilent, Oxford) with graphite-monochromator Mo-Kα ($\lambda$ = 0.71073 Å) at about 110 K. Powder X-ray diffraction (PXRD) patterns were collected on a Bruker X-ray diffractometer (D8 ADVANCE X) using Cu-Kα radiation. The gas adsorption isotherms were collected on an automatic volumetric adsorption apparatus of BSD-PS (M) (Specific surface area & pore size analyzer). The breakthrough experiments were carried out in a dynamic gas breakthrough of BSD-MAB (Multi-component Adsorption Breakthrough Curve Analyzer). Differential scanning calorimetry (DSC) characterizations were conducted on a NETZSCH DSC214 instrument.

**Preparation of SPOC-SQ-a microcrystalline powder**

The microcrystalline powder of SPOC-SQ-DCM was obtained by slow evaporation in a saturated DCM solution of SPOC-SQ, and then activated in air under room temperature for at least 6 h, generating the SPOC-SQ-a microcrystalline powder.

**Single-component gas adsorption of SPOC-SQ-a microcrystalline for C1, C2, and C3 hydrocarbons**

The gas adsorption isotherms were collected on an automatic volumetric adsorption apparatus BSD-PS (M) (Specific surface area & pore size analyzer). Before the adsorption measurements, the as-prepared sample (SPOC-SQ-a) was dried under high vacuum for 24 h at 60 °C for gas adsorption analyses. The different temperatures for gas adsorption measurements were maintained by using ice salt bath, ice bath, and water bath. After gas adsorption measurements were completed, the sample was evacuated by a vacuum pump for 6 h at 60 °C until the pressure was below 0.1 bar to ensure complete removal of adsorbent.

**Gate-opening enthalpy**

The Clausius-Clapeyron equation was used to calculate the molar enthalpy of gate opening ($\Delta H_{GO}$).

$d\ln P_{GO}/(d(1/T)) = \Delta H_{GO}/\mathrm{R}$

$P_{GO}$ is gate opening pressure, $T$ is measurement temperature, R is gas constant (8.314 J $mol^{-1}$ $K^{-1}$).

**Preparation of SPOC-SQ-a single crystal**

The single crystal of SPOC-SQ-a was easily obtained by slow removal of DCM from SPOC-SQ-DCM single crystal in air at room temperature.

**Preparation of SPOC-SQ-$CO_2$ and SPOC-SQ-$CO_2$-MO single crystals**

Single crystal of SPOC-SQ-$CO_2$ was obtained by placing SPOC-SQ-a single crystal in $CO_2$ gas atmosphere at about 1 bar under 203 K (acetone and liquid nitrogen) for about 12 h. SPOC-SQ-$CO_2$ single crystal could be transformed to SPOC-SQ-$CO_2$-MO single crystal upon vacuum treatment at room temperature for 1 h, which could be further activated back to SPOC-SQ-a by vacuuming at 60 °C for 3 h.

**Preparation of SPOC-SQ-$C_2H_2$ and SPOC-SQ-$C_2H_2$-MO single crystals**

Single crystal of SPOC-SQ-$C_2H_2$ was obtained by placing SPOC-SQ-a single crystal in $C_2H_2$ gas atmosphere at about 1 bar under 298 K for about 12 h. SPOC-SQ-$C_2H_2$ single crystal could be transformed to SPOC-SQ-$C_2H_2$-MO single crystal upon vacuum treatment at room temperature for more than 30 min, which could be further activated back to SPOC-SQ-a by vacuuming at 60 °C for 3 h.

**Preparation of SPOC-SQ-$C_2H_4$, SPOC-SQ-$C_2H_4$-MO, and SPOC-SQ-$C_2H_4$-MSO single crystals**

Single crystal of SPOC-SQ-$C_2H_4$ was obtained by placing SPOC-SQ-a single crystal in $C_2H_4$ gas atmosphere at about 1 bar under 253 K (ice salt bath: 500 g crushed ice and 164.89 g sodium chloride) for about 12 h. SPOC-SQ-$C_2H_4$ single crystal could be transformed to SPOC-SQ-$C_2H_4$-MO and SPOC-SQ-$C_2H_4$-MSO single crystals upon treatment in air at room temperature for about 5 and 60 min, respectively, which could be further activated back to SPOC-SQ-a by vacuuming at 60 °C for 3 h.

**Preparation of SPOC-SQ-$C_2H_6$, SPOC-SQ-$C_2H_6$-MO, and SPOC-SQ-$C_2H_6$-MSO single crystals**

Single crystal of SPOC-SQ-$C_2H_6$ was obtained by placing SPOC-SQ-a single crystal in $C_2H_6$ gas atmosphere at about 1 bar under 253 K (ice salt bath: 500 g crushed ice and 164.89 g sodium chloride) for about 12 h. SPOC-SQ-$C_2H_6$ single crystal could be transformed to SPOC-SQ-$C_2H_6$-MO and SPOC-SQ-$C_2H_6$-MSO single crystals upon treatment in air at room temperature for about 20 s and 30 min, respectively, which could be further activated back to SPOC-SQ-a in air for more than 3 h.

**Preparation of SPOC-SQ-$C_3H_4$ single crystal**

Single crystal of SPOC-SQ-$C_3H_4$ was obtained by placing SPOC-SQ-a single crystal in $C_3H_4$ gas atmosphere at about 1 bar under 273 K for about 12 h. SPOC-SQ-$C_3H_4$ single crystal could be activated back to SPOC-SQ-a by vacuuming at 60 °C for 3 h.

**Preparation of SPOC-SQ-$C_3H_6$ and SPOC-SQ-$C_3H_6$-MO single crystals**

Single crystal of SPOC-SQ-$C_3H_6$ was obtained by placing SPOC-SQ-a single crystal in $C_3H_6$ gas atmosphere at about 1 bar under 273 K for about 12 h. SPOC-SQ-$C_3H_6$ singe crystal could be transformed to SPOC-SQ-$C_2H_4$-MO single crystals upon treatment in air at room temperature for about 40 s, which could be further activated back to SPOC-SQ-a in air for more than 30 min.

**Single crystal X-ray crystallographic resolution**

The structures were solved by the direct method and refined by the full-matrix least-squares method on $F^2$ with anisotropic thermal parameters for all non-hydrogen atoms. Gas molecules of $CO_2$, $C_2H_2$, $C_2H_4$, $C_2H_6$, $C_3H_4$, and $C_3H_6$ were located from enough electron density peaks

and refined with isotropic displacement parameters. Due to the gas molecular movements inside the SPOC-SQ frameworks, the crystallographic analysis was carried out through reasonable refinements to ensure their positions, the resolved results met the normal bond lengths (1.174 Å of C-O for $CO_2$, 1.136 Å, 1.298 Å, and 1.591 Å for C-C in $C_2H_2$, $C_2H_4$, and $C_2H_6$, respectively, 1.174 Å and 1.466 Å for C-C in $C_3H_4$, and 1.309 Å and 1.514 Å for C-C in $C_3H_6$). Hydrogen atoms on $C_2H_2$, $C_2H_4$, $C_2H_6$, $C_3H_4$, and $C_3H_6$ molecules were added with geometrical constraints. Crystallographic data and structure refinement data were shown in Tables S2-S7. Further supplementary crystallographic data for the above single crystals can be obtained free of charge from the Cambridge Crystallographic Data Centre via www.ccdc.cam.ac.uk/data_request/cif (CCDC number: 2220252-2220269).

**Computational methods**

The first-principles calculations were carried out within the framework of density functional theory (DFT) within the general gradient approximation (GGA) by Perdew-Burke-Ernzerhof (PBE) exchange-correlation functional using the QUICKSTEP module in the CP2K package.[1] Double-zeta valence plus polarization (DZVP) basis sets was employed, combined through norm-conserving Goedecker-Teter-Hutter (GTH) pseudo potentials[2-3] and the Gaussian and Augmented Plane Wave (GAPW) hybrid basis set.[4] The DFTD3 correction[5] was included in the calculation to fully capture the long-range dispersion interaction between the framework of SPOC-SQ-gas and gas molecules. The crystal and adsorption structures were fully relaxed until the maximum atomic force was less than $2 \times 10^{-2}$ eV $Å^{-1}$.

**PXRD analysis of microcrystalline powders**

PXRD patterns were collected by a Bruker X-ray diffractometer using Cu-Kα radiation. The data were collected in the range of $2\theta$ = 5° - 45°. In-situ PXRD patterns were collected at different time after placing SPOC-SQ-gas in air for 12 hours. (Gases: $CO_2$ and $C_2H_2$)

**DSC for SPOC-SQ-$CO_2$-MO and SPOC-SQ-$C_2H_2$-MO crystals**

DSC analyses were conducted on a NETZSCH DSC214 instrument. Single crystals of SPOC-SQ-$CO_2$-MO and SPOC-SQ-$C_2H_2$-MO obtained after removal of $CO_2$ and $C_2H_2$ from SPOC-SQ-$CO_2$ and SPOC-SQ-$C_2H_2$ were placed in Tzero aluminum pans and heated from 273 K to the 373 K at a rate of 5, 10, or15 K min$^{-1}$.

**Single-component gas adsorptions for MSME of SPOC-SQ-$CO_2$-MO and SPOC-SQ-$C_2H_2$-MO**

The gas adsorption isotherms were collected on an automatic volumetric adsorption apparatus BSD-PS (M) (Specific surface area & pore size analyzer). Before the adsorption measurements, the as-synthesized sample (SPOC-SQ-DCM) was dried under high vacuum for 24 h at 60 °C to remove the DCM molecules, giving the activated SPOC-SQ-a for gas adsorption analyses. Gas adsorption tests for $CO_2$ and $C_2H_2$ were maintained at 253 K and 298 K by using ice salt bath and water bath, respectively. The second to fifth cycle of absorption tests were performed without activation.

**Isotherm data were analyzed using the virial equation**

$$\ln(n/P) = A_0 + A_1 n + A_2 n^2 + \cdots$$

where $P$ is pressure, n is the amount adsorbed, and $A_0$, $A_1$, $A_2$, etc., are virial coefficients. $A_0$ is related to adsorbate-adsorbent interactions, whereas $A_1$ describes adsorbate-adsorbate interactions. The Henry's law constant ($K_H$) is equal to exp ($A_0$), and the selectivity can be obtained from $K_H$.

**Breakthrough separation experiments for $C_3H_6$/$C_3H_8$ (*v*/*v*, 50/50) separation**

In a typical two-gas breakthrough experiment, 4.2 g of pre-activated, tandem-packed sample was placed in quartz tubing (10 mm diameter) to form a fixed bed. The adsorbent bed was purged under a 60 cm$^3$/min flow of He gas at 60 °C for 2 h before the breakthrough experiment. Upon cooling to 0 °C, a 5 cm$^3$/min gas mixture containing 50% $C_3H_6$ and 50% $C_3H_8$ was introduced. The outlet composition was continuously monitored by BSD-MS until a complete

breakthrough was achieved. When the outlet composition of two gases reached equilibrium, gas mixture flow was then shut off.

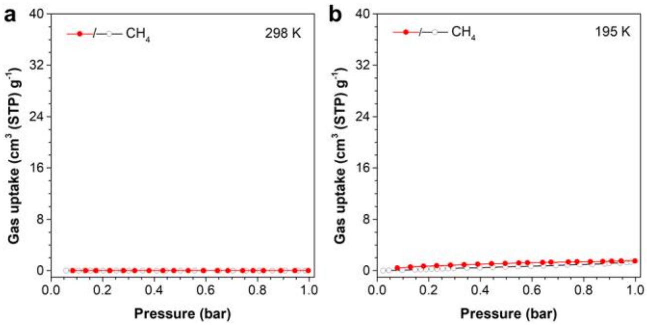


**Figure S1**. Sorption isotherms of SPOC-SQ-a towards $CH_4$ at (a) 298 K and (b) 195 K. Filled and open circle symbols represent adsorption and desorption, respectively.

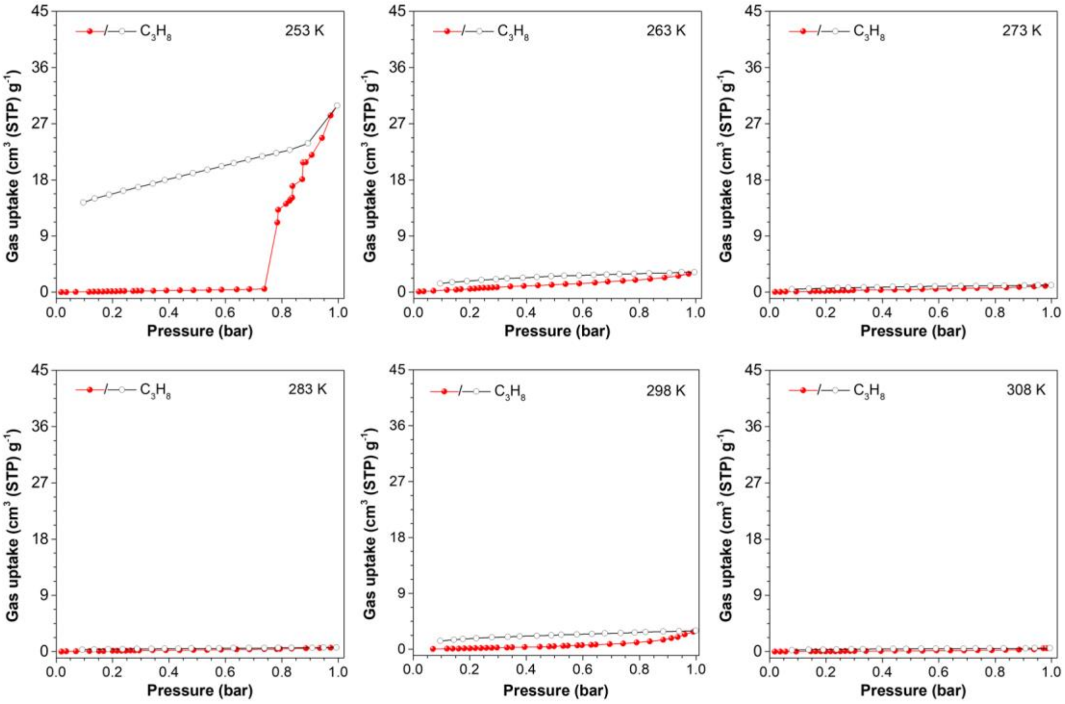


**Figure S2**. Sorption isotherms of SPOC-SQ-a towards $C_3H_8$ at 253 K, 263 K, 273 K, 283 K, 298 K, and 308 K. Filled and open circle symbols represent adsorption and desorption, respectively.

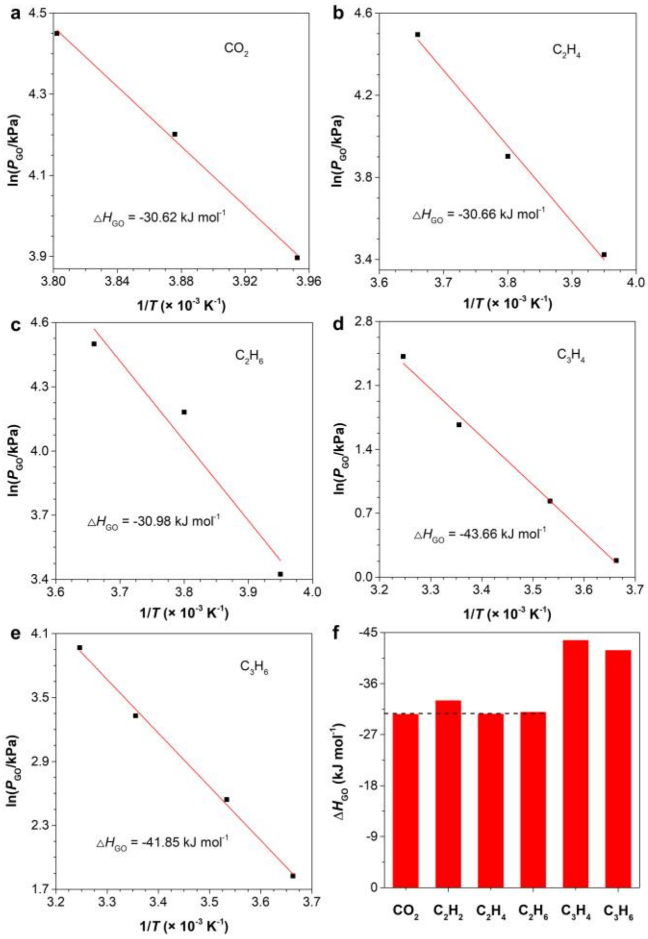


**Figure S3**. Plots of gate-opening pressure ($\ln P_{GO}$) vs inverse of measurement temperature ($1/T$) for adsorption isotherms of (a) $CO_2$, (b) $C_2H_4$, (c) $C_2H_6$, (d) $C_3H_4$, and (e) $C_3H_6$. (f) Comparison of their enthalpies of gate opening ($\Delta H_{GO}$) obtained according to the Clausius-Clapeyron equation: $d\ln P_{GO}/(d(1/T)) = \Delta H_{GO}/R$.

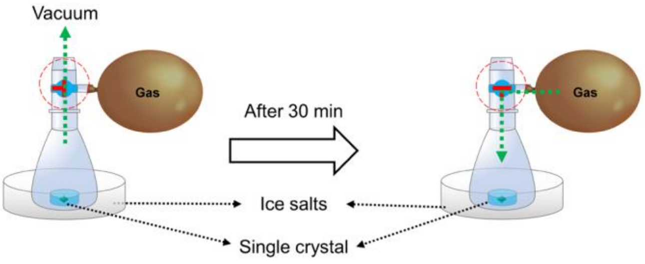


**Figure S4**. Schematic illustration of the apparatus used for the single crystal growth in gas atmosphere at low temperature.

**Table S1**. Single crystal growth conditions under gas atmosphere.

| **Gas** | **$CO_2$** | **$C_2H_2$** | **$C_2H_4$** | **$C_2H_6$** | **$C_3H_4$** | **$C_3H_6$** |
|---|---|---|---|---|---|---|

| Temperature (K) | 203 | 298 | 253 | 253 | 273 | 273 |
|---|---|---|---|---|---|---|
| **Pressure (bar)** | 1 | 1 | 1 | 1 | 1 | 1 |

**Table S2**. Crystal data and structure refinement of SPOC-SQ-$CO_2$, SPOC-SQ-$CO_2$-MO, and SPOC-SQ-a after $CO_2$ removal.

| Name | SPOC-SQ-$CO_2$ | SPOC-SQ-$CO_2$-MO | SPOC-SQ-a after $CO_2$ removal |
|---|---|---|---|
| CCDC number | 2220262 | 2220260 | 2220269 |
| Empirical formula | $C_{28.14394}H_{16}Cl_4N_2O_{2.28788}$ | $C_{28}H_{16}Cl_4N_2O_2$ | $C_{28}H_{16}Cl_4N_2O_2$ |
| Formula weight | 560.56 | 554.23 | 554.23 |
| Temperature/K | 122(4) | 126(12) | 120.0(6) |
| Crystal system | triclinic | triclinic | triclinic |
| Space group | P-1 | P-1 | P-1 |
| *a*/Å | 8.725(4) | 8.701(6) | 12.8242(18) |
| *b*/Å | 8.867(4) | 8.844(12) | 13.763(2) |
| *c*/Å | 9.610(7) | 9.591(15) | 16.591(3) |
| $\alpha$/° | 115.92(6) | 116.14(15) | 110.088(14) |
| $\beta$/° | 94.03(5) | 93.91(9) | 111.009(14) |
| $\gamma$/° | 92.15(4) | 92.69(9) | 91.101(12) |
| *Volume*/Å$^3$ | 665.1(7) | 658.5(15) | 2533.2(7) |
| *Z* | 1 | 1 | 4 |
| $\rho_{calc}$mg/mm$^3$ | 1.399 | 1.398 | 1.453 |
| *m*/mm$^{-1}$ | 0.475 | 0.478 | 0.497 |
| *F*(000) | 285 | 282 | 1128 |
| Crystal size/mm$^3$ | 0.24 × 0.17 × 0.13 | 0.12 × 0.11 × 0.09 | 0.16 × 0.12 × 0.06 |
| 2$\Theta$ range for data collection | 6.68 to 51.98° | 6.68 to 52° | 5.94 to 48° |
| Index ranges | -10 ≤ h ≤ 9, -10 ≤ k ≤ 10, -11 ≤ l ≤ 11 | -10 ≤ h ≤ 9, -10 ≤ k ≤ 8, -9 ≤ l ≤ 11 | -14 ≤ h ≤ 14, -14 ≤ k ≤ 15, -17 ≤ l ≤ 18 |
| Reflections collected | 4178 | 3704 | 15254 |
| Independent reflections | 2556[R(int) = 0.1127 (inf-0.9Å)] | 2477[R(int) = 0.2016 (inf-0.9Å)] | 7919[R(int) = 0.1555 (inf-0.9Å)] |
| Data/restraints/parameters | 2556/13/173 | 2477/152/139 | 7919/432/649 |

| | | | |
|---|---|---|---|
| Goodness-of-fit on $F^2$ | 1.037 | 0.971 | 1.017 |
| Final *R* indexes [$I>=2\sigma (I)$] | $R_1$ = 0.1178, w$R_2$ = 0.2284 | $R_1$ = 0.1284, w$R_2$ = 0.2258 | $R_1$ = 0.1100, w$R_2$ = 0.1930 |
| Final *R* indexes [all data] | $R_1$ = 0.2309, w$R_2$ = 0.3000 | $R_1$ = 0.4068, w$R_2$ = 0.3617 | $R_1$ = 0.2652, w$R_2$ = 0.2679 |
| Largest diff. peak/hole / e Å$^{-3}$ | 0.647/-0.389 | 0.382/-0.336 | 1.453/-0.349 |
| Flack Parameters | N | N | N |
| Completeness | 0.9916 | 0.9732 | 0.9957 |

$R_1 = \Sigma||F_o| - |F_c||/\Sigma|F_o|$, $wR_2 = [\Sigma w(F_o^2 - F_c^2)^2/\Sigma w(F_o^2)^2]^{1/2}$

**Table S3.** Crystal data and structure refinement of SPOC-SQ-$C_2H_2$, SPOC-SQ-$C_2H_2$-MO, and SPOC-SQ-a after $C_2H_2$ removal.

| Name | SPOC-SQ-$C_2H_2$ | SPOC-SQ-$C_2H_2$-MO | SPOC-SQ-a after $C_2H_2$ removal |
|---|---|---|---|
| CCDC number | 2034380 | 2220253 | 2220252 |
| Empirical formula | $C_{30.10044}H_{18.10044}Cl_4N_2O_2$ | $C_{28}H_{16}Cl_4N_2O_2$ | $C_{28}H_{16}Cl_4N_2O_2$ |
| Formula weight | 581.57 | 554.23 | 554.23 |
| Temperature/K | 119(5) | 113.40(14) | 106.0(10) |
| Crystal system | triclinic | triclinic | triclinic |
| Space group | P-1 | P-1 | P-1 |
| *a*/Å | 8.7325(10) | 8.690(14) | 12.8312(18) |
| *b*/Å | 8.8710(9) | 8.914(13) | 13.764(2) |
| *c*/Å | 9.6585(14) | 9.585(17) | 16.547(2) |
| $\alpha$/° | 94.715(10) | 116.08(15) | 110.050(13) |
| $\beta$/° | 115.018(13) | 93.95(14) | 111.046(13) |
| $\gamma$/° | 90.156(9) | 92.71(12) | 91.168(12) |
| *Volume*/Å$^3$ | 675.13(14) | 662.77(18) | 2527.5(6) |
| *Z* | 1 | 1 | 4 |
| $\rho_{calc}$mg/mm$^3$ | 1.430 | 1.389 | 1.457 |
| *m*/mm$^{-1}$ | 0.470 | 0.475 | 0.498 |
| *F*(000) | 297 | 282 | 1128 |
| Crystal size/mm$^3$ | 0.34 × 0.32 × 0.23 | 0.22 × 0.18 × 0.12 | 0.28 × 0.26 × 0.12 |
| $2\Theta$ range for data collection | 6.26 to 51.98° | 7 to 52° | 6.36 to 52° |
| Index ranges | $-10 \le h \le 10$, $-10 \le k \le 9$, | $-10 \le h \le 10$, $-10 \le k \le 10$, | $-14 \le h \le 15$, $-16 \le k \le 16$, |

| | -11 ≤ l ≤ 9 | -9 ≤ l ≤ 11 | -20 ≤ l ≤ 19 |
|---|---|---|---|
| Reflections collected | 4542 | 4178 | 16995 |
| Independent reflections | 2639[R(int) = 0.0404 (inf-0.9Å)] | 2524[R(int) = 0.0513 (inf-0.9Å)] | 9574[R(int) = 0.1439 (inf-0.9Å)] |
| Data/restraints/parameters | 2639/2/183 | 2524/0/163 | 9574/584/649 |
| Goodness-of-fit on $F^2$ | 1.093 | 1.045 | 0.980 |
| Final $R$ indexes [$I$>=2$\sigma$ ($I$)] | $R_1$ = 0.0597, w$R_2$ = 0.1288 | $R_1$ = 0.0615, w$R_2$ = 0.1158 | $R_1$ = 0.1081, w$R_2$ = 0.1694 |
| Final $R$ indexes [all data] | $R_1$ = 0.0852, w$R_2$ = 0.1436 | $R_1$ = 0.1041, w$R_2$ = 0.1435 | $R_1$ = 0.2633, w$R_2$ = 0.2414 |
| Largest diff. peak/hole/eÅ$^{-3}$ | 0.309/-0.283 | 0.436/-0.390 | 1.091/-0.374 |
| Flack Parameters | N | N | N |
| Completeness | 0.9949 | 0.9873 | 0.9939 |

$R_1 = \Sigma||F_o| - |F_c||/\Sigma|F_o|$, $wR_2 = [\Sigma w(F_o^2 - F_c^2)^2/\Sigma w(F_o^2)^2]^{1/2}$

**Table S4.** Crystal data and structure refinement of SPOC-SQ-$C_2H_4$, SPOC-SQ-$C_2H_4$-MO, SPOC-SQ-$C_2H_4$-MSO, and SPOC-SQ-a after $C_2H_4$ removal.

| Name | SPOC-SQ-$C_2H_4$ | SPOC-SQ-$C_2H_4$-MO | SPOC-SQ-$C_2H_4$-MSO | SPOC-SQ-a after $C_2H_4$ removal |
|---|---|---|---|---|
| CCDC number | 2220258 | 2220254 | 2220255 | 2220265 |
| Empirical formula | $C_{29.10898}H_{18.21796}Cl_4N_2O_2$ | $C_{28}H_{16}Cl_4N_2O_2$ | $C_{28}H_{16}Cl_4N_2O_2$ | $C_{28}H_{16}Cl_4N_2O_2$ |
| Formula weight | 569.78 | 554.23 | 554.23 | 554.23 |
| Temperature/K | 116(2) | 116(3) | 114.55(10) | 113.3(2) |
| Crystal system | triclinic | triclinic | triclinic | triclinic |
| Space group | P-1 | P-1 | P-1 | P-1 |
| $a$/Å | 8.7378(12) | 8.733(2) | 9.314(11) | 12.826(4) |
| $b$/Å | 8.8280(14) | 8.842(2) | 9.507(6) | 13.793(3) |
| $c$/Å | 9.6641(17) | 9.538(2) | 14.399(9) | 16.617(4) |
| $\alpha$/° | 93.944(14) | 115.68(2) | 87.70(6) | 110.09(2) |
| $\beta$/° | 115.342(15) | 93.96(2) | 85.83(8) | 111.15(3) |
| $\gamma$/° | 91.202(12) | 92.18(2) | 86.04(7) | 90.96(2) |
| $Volume$/Å$^3$ | 671.08(18) | 660.2(3) | 1267.8(19) | 2541.5(11) |
| $Z$ | 1 | 1 | 2 | 4 |
| $\rho_{calc}$mg/mm$^3$ | 1.410 | 1.394 | 1.452 | 1.448 |
| $m$/mm$^{-1}$ | 0.471 | 0.477 | 0.497 | 0.495 |

| $F(000)$ | 291 | 282 | 564 | 1128 |
|---|---|---|---|---|
| Crystal size/mm$^3$ | 0.34 × 0.23 × 0.11 | 0.28 × 0.24 × 0.22 | 0.32 × 0.23 × 0.18 | 0.35 × 0.34 × 0.27 |
| $2\Theta$ range for data collection | 6.74 to 51.98° | 6.36 to 45.98° | 5.94 to 51.98° | 6.74 to 52° |
| Index ranges | -10 ≤ h ≤ 10, -10 ≤ k ≤ 9, -11 ≤ l ≤ 11 | -9 ≤ h ≤ 9, -9 ≤ k ≤ 9, -9 ≤ l ≤ 10 | -11 ≤ h ≤ 11, -9 ≤ k ≤ 11, -17 ≤ l ≤ 15 | -14 ≤ h ≤ 15, -17 ≤ k ≤ 16, -20 ≤ l ≤ 20 |
| Reflections collected | 4306 | 3329 | 8774 | 17355 |
| Independent reflections | 2613[R(int) = 0.0517 (inf-0.9Å)] | 1824[R(int) = 0.0734 (inf-0.9Å)] | 4872[R(int) = 0.2131 (inf-0.9Å)] | 9766[R(int) = 0.1089 (inf-0.9Å)] |
| Data/restraints/parameters | 2613/7/173 | 1824/146/163 | 4872/615/413 | 9766/420/649 |
| Goodness-of-fit on $F^2$ | 1.102 | 1.095 | 1.022 | 1.214 |
| Final $R$ indexes [$I$>=2$\sigma$ ($I$)] | $R_1$ = 0.0743, w$R_2$ = 0.1540 | $R_1$ = 0.0833, w$R_2$ = 0.2239 | $R_1$ = 0.1735, w$R_2$ = 0.3428 | $R_1$ = 0.1645, w$R_2$ = 0.3660 |
| Final $R$ indexes [all data] | $R_1$ = 0.1116, w$R_2$ = 0.1803 | $R_1$ = 0.1283, w$R_2$ = 0.2584 | $R_1$ = 0.4401, w$R_2$ = 0.5069 | $R_1$ = 0.2598, w$R_2$ = 0.4550 |
| Largest diff. peak/hole / e Å$^{-3}$ | 0.396/-0.494 | 1.016/-0.341 | 0.459/-0.403 | 3.058/-0.623 |
| Flack Parameters | N | N | N | N |
| Completeness | 0.9909 | 0.9864 | 0.9905 | 0.9772 |

$R_1 = \Sigma||F_o| - |F_c||/\Sigma|F_o|$, $wR_2 = [\Sigma w(F_o^2 - F_c^2)^2/\Sigma w(F_o^2)^2]^{1/2}$

**Table S5.** Crystal data and structure refinement of SPOC-SQ-$C_2H_6$, SPOC-SQ-$C_2H_6$-MO, SPOC-SQ-$C_2H_6$-MSO, and SPOC-SQ-a after $C_2H_6$ removal.

| Name | SPOC-SQ-$C_2H_6$ | SPOC-SQ-$C_2H_6$-MO | SPOC-SQ-$C_2H_6$-MSO | SPOC-SQ-a after $C_2H_6$ removal |
|---|---|---|---|---|
| CCDC number | 2220268 | 2220264 | 2220266 | 2220267 |
| Empirical formula | $C_{29.85152}H_{21.55456}Cl_4N_2O_2$ | $C_{28}H_{16}Cl_4N_2O_2$ | $C_{28}H_{16}Cl_4N_2O_2$ | $C_{28}H_{16}Cl_4N_2O_2$ |
| Formula weight | 582.06 | 554.23 | 554.23 | 554.23 |
| Temperature/K | 119(7) | 113.20(14) | 115(2) | 113.45(10) |
| Crystal system | triclinic | triclinic | triclinic | triclinic |
| Space group | P-1 | P-1 | P-1 | P-1 |
| $a$/Å | 8.064(3) | 8.739(4) | 9.287(4) | 12.831(3) |
| $b$/Å | 9.307(3) | 8.822(4) | 9.482(4) | 13.765(3) |
| $c$/Å | 9.775(4) | 9.625(5) | 14.333(5) | 16.563(4) |

| | | | | |
|---|---|---|---|---|
| $\alpha$/° | 86.73(3) | 93.92(4) | 87.69(3) | 110.06(2) |
| $\beta$/° | 68.69(3) | 115.31(5) | 85.95(3) | 111.036(19) |
| $\gamma$/° | 86.29(3) | 91.50(4) | 85.88(3) | 91.065(17) |
| *Volume*/Å$^3$ | 681.5(4) | 668.0(5) | 1254.9(8) | 2531.1(9) |
| *Z* | 1 | 1 | 2 | 4 |
| $\rho_{calc}$mg/mm$^3$ | 1.418 | 1.378 | 1.467 | 1.454 |
| *m*/mm$^{-1}$ | 0.466 | 0.471 | 0.502 | 0.498 |
| *F*(000) | 299 | 282 | 564 | 1128 |
| Crystal size/mm$^3$ | 0.55 × 0.48 × 0.43 | 0.43 × 0.41 × 0.40 | 0.23 × 0.22 × 0.21 | 0.32 × 0.32 × 0.15 |
| 2$\Theta$ range for data collection | 6.82 to 51.98° | 6.88 to 51.98° | 6.96 to 52° | 6.74 to 52° |
| Index ranges | -7 ≤ h ≤ 9, -10 ≤ k ≤ 11, -12 ≤ l ≤ 11 | -9 ≤ h ≤ 10, -10 ≤ k ≤ 10, -11 ≤ l ≤ 11 | -11 ≤ h ≤ 11, -7 ≤ k ≤ 11, -15 ≤ l ≤ 17 | -15 ≤ h ≤ 15, -16 ≤ k ≤ 16, -18 ≤ l ≤ 20 |
| Reflections collected | 4452 | 4057 | 6627 | 17818 |
| Independent reflections | 2607[R(int) = 0.1153 (inf-0.9Å)] | 2535[R(int) = 0.1245 (inf-0.9Å)] | 4637[R(int) = 0.1353 (inf-0.9Å)] | 9589[R(int) = 0.1559 (inf-0.9Å)] |
| Data/restraints/parameters | 2607/0/174 | 2535/102/163 | 4637/633/401 | 9589/414/649 |
| Goodness-of-fit on $F^2$ | 1.079 | 1.183 | 1.011 | 1.032 |
| Final *R* indexes [$I$>=2$\sigma$ ($I$)] | $R_1$ = 0.1385, w$R_2$ = 0.3236 | $R_1$ = 0.1688, w$R_2$ = 0.3575 | $R_1$ = 0.1536, w$R_2$ = 0.3419 | $R_1$ = 0.1373, w$R_2$ = 0.2836 |
| Final *R* indexes [all data] | $R_1$ = 0.2047, w$R_2$ = 0.3931 | $R_1$ = 0.2819, w$R_2$ = 0.4323 | $R_1$ = 0.3689, w$R_2$ = 0.5030 | $R_1$ = 0.3314, w$R_2$ = 0.4074 |
| Largest diff. peak/hole / e Å$^{-3}$ | 1.178/-0.684 | 0.894/-0.516 | 0.719/-0.819 | 1.523/-0.451 |
| Flack Parameters | N | N | N | N |
| Completeness | 0.9719 | 0.9870 | 0.9642 | 0.9865 |

$R_1 = \Sigma||F_o| - |F_c||/\Sigma|F_o|$, $wR_2 = [\Sigma w(F_o^2 - F_c^2)^2/\Sigma w(F_o^2)^2]^{1/2}$

**Table S6.** Crystal data and structure refinement of crystals SPOC-SQ-$C_3H_4$ and SPOC-SQ-a after $C_3H_4$ removal.

| Name | SPOC-SQ-$C_3H_4$ | SPOC-SQ-a after $C_3H_4$ removal |
|---|---|---|
| CCDC number | 2220257 | 2220261 |
| Empirical formula | $C_{31}H_{20}Cl_4N_2O_2$ | $C_{28}H_{16}Cl_4N_2O_2$ |
| Formula weight | 594.29 | 554.23 |
| Temperature/K | 114(4) | 111.55(10) |
| Crystal system | triclinic | triclinic |
| Space group | P-1 | P-1 |
| $a$/Å | 6.9024(19) | 12.870(3) |
| $b$/Å | 9.767(3) | 13.804(4) |
| $c$/Å | 10.398(3) | 16.626(5) |
| $\alpha$/° | 103.85(2) | 109.91(3) |
| $\beta$/° | 102.38(2) | 111.03(2) |
| $\gamma$/° | 91.68(2) | 91.26(2) |
| *Volume*/Å$^3$ | 662.4(3) | 2556.9(12) |
| $Z$ | 1 | 4 |
| $\rho_{calc}$mg/mm$^3$ | 1.490 | 1.440 |
| $m$/mm$^{-1}$ | 0.481 | 0.492 |
| $F$(000) | 304 | 1128 |
| Crystal size/mm$^3$ | 0.35 × 0.34 × 0.13 | 0.31 × 0.23 × 0.22 |
| 2$\Theta$ range for data collection | 6.7 to 52° | 5.92 to 48° |
| Index ranges | $-7 \le h \le 8$, $-11 \le k \le 12$, $-12 \le l \le 11$ | $-14 \le h \le 12$, $-15 \le k \le 15$, $-13 \le l \le 19$ |
| Reflections collected | 4029 | 12604 |
| Independent reflections | 2527[R(int) = 0.0789 (inf-0.9Å)] | 7760[R(int) = 0.1713 (inf-0.9Å)] |
| Data/restraints/parameters | 2527/24/191 | 7760/584/649 |
| Goodness-of-fit on $F^2$ | 1.052 | 1.038 |
| Final $R$ indexes [$I$>=2$\sigma$ ($I$)] | $R_1$ = 0.1050, w$R_2$ = 0.2427 | $R_1$ = 0.1389, w$R_2$ = 0.2944 |
| Final $R$ indexes [all data] | $R_1$ = 0.1665, w$R_2$ = 0.2975 | $R_1$ = 0.3133, w$R_2$ = 0.4402 |
| Largest diff. peak/hole / e Å$^{-3}$ | 1.013/-0.565 | 1.091/-0.504 |
| Flack Parameters | N | N |
| Completeness | 0.9836 | 0.9638 |

$R_1 = \Sigma||F_o| - |F_c||/\Sigma|F_o|$, $wR_2 = [\Sigma w(F_o^2 - F_c^2)^2/\Sigma w(F_o^2)^2]^{1/2}$

**Table S7.** Crystal data and structure refinement of crystals SPOC-SQ-$C_3H_6$, SPOC-SQ-$C_3H_6$-MO, and SPOC-SQ-a after $C_3H_6$ removal.

| Name | SPOC-SQ-$C_3H_6$ | SPOC-SQ-$C_3H_6$-MO | SPOC-SQ-a after $C_3H_6$ removal |
|---|---|---|---|
| CCDC number | 2220256 | 2220263 | 2220259 |
| Empirical formula | $C_{31}H_{22}Cl_4N_2O_2$ | $C_{28}H_{16}Cl_4N_2O_2$ | $C_{56}H_{32}Cl_8N_4O_4$ |
| Formula weight | 596.31 | 554.23 | 1108.46 |
| Temperature/K | 121(6) | 112.2(14) | 118.05(10) |
| Crystal system | triclinic | triclinic | triclinic |
| Space group | P-1 | P-1 | P-1 |
| $a$/Å | 8.530(3) | 9.121(11) | 12.862(4) |
| $b$/Å | 9.049(3) | 9.918(7) | 13.775(4) |
| $c$/Å | 9.934(3) | 15.597(11) | 16.600(6) |
| $\alpha$/° | 94.20(3) | 79.67(6) | 110.12(3) |
| $\beta$/° | 115.28(4) | 86.30(8) | 111.23(3) |
| $\gamma$/° | 91.16(3) | 85.92(8) | 90.97(3) |
| *Volume*/Å$^3$ | 690.4(4) | 1383(2) | 2540.4(14) |
| $Z$ | 1 | 2 | 2 |
| $\rho_{calc}$mg/mm$^3$ | 1.434 | 1.331 | 1.449 |
| $m$/mm$^{-1}$ | 0.462 | 0.455 | 0.496 |
| $F$(000) | 306 | 564 | 1128 |
| Crystal size/mm$^3$ | 0.14 × 0.14 × 0.13 | 0.24 × 0.21 × 0.13 | 0.20 × 0.18 × 0.11 |
| 2$\Theta$ range for data collection | 6.76 to 52° | 5.94 to 52° | 6.34 to 52° |
| Index ranges | $-10 \leq h \leq 10$, $-10 \leq k \leq 11$, $-10 \leq l \leq 12$ | $-11 \leq h \leq 10$, $-9 \leq k \leq 12$, $-19 \leq l \leq 18$ | $-15 \leq h \leq 11$, $-16 \leq k \leq 16$, $-20 \leq l \leq 20$ |
| Reflections collected | 4202 | 8460 | 18986 |
| Independent reflections | 2660[R(int) = 0.0689 (inf-0.9Å)] | 5210[R(int) = 0.2088 (inf-0.9Å)] | 9730[R(int) = 0.1679 (inf-0.9Å)] |
| Data/restraints/parameters | 2660/19/191 | 5210/334/265 | 9730/420/649 |
| Goodness-of-fit on $F^2$ | 1.096 | 1.466 | 1.026 |
| Final $R$ indexes [$I$>=2$\sigma$ ($I$)] | $R_1$ = 0.0981, w$R_2$ = 0.1784 | $R_1$ = 0.2880, w$R_2$ = 0.5844 | $R_1$ = 0.1390, w$R_2$ = 0.3104 |
| Final $R$ indexes [all data] | $R_1$ = 0.1729, w$R_2$ = 0.2216 | $R_1$ = 0.4898, w$R_2$ = 0.6500 | $R_1$ = 0.3354, w$R_2$ = 0.4257 |
| Largest diff. peak/hole/eÅ$^{-3}$ | 0.533/-0.342 | 1.751/-0.755 | 1.289/-0.487 |

| | | | |
|---|---|---|---|
| Flack Parameters | N | N | N |
| Completeness | 0.9770 | 0.9727 | 0.9939 |

$R_1 = \Sigma||F_o| - |F_c||/\Sigma|F_o|$, $wR_2 = [\Sigma w(F_o^2 - F_c^2)^2/\Sigma w(F_o^2)^2]^{1/2}$

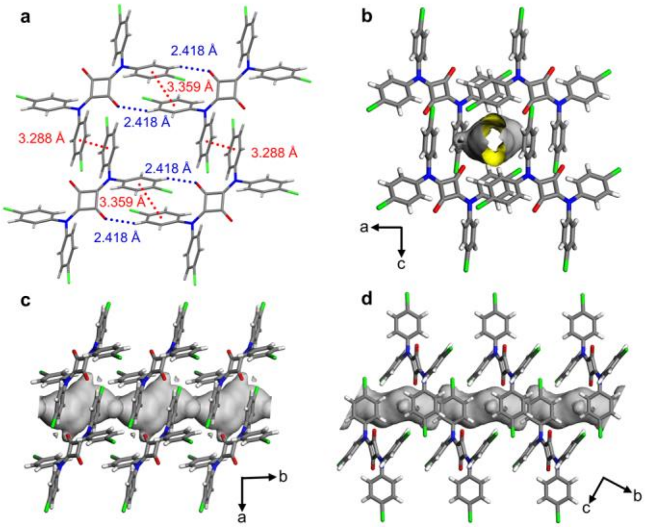


**Figure S5**. (a) The intermolecular interactions between SPOC-SQ molecules for the framework of SPOC-SQ-$CO_2$. (b-d) Packing diagrams of SPOC-SQ-$CO_2$ with the solvent-accessible void space visualized by yellow/grey (inner/outer) curved planes generated with a probe of 1.4 Å. Gray, blue, red, green, and white represent C, N, O, Cl, and H, respectively.

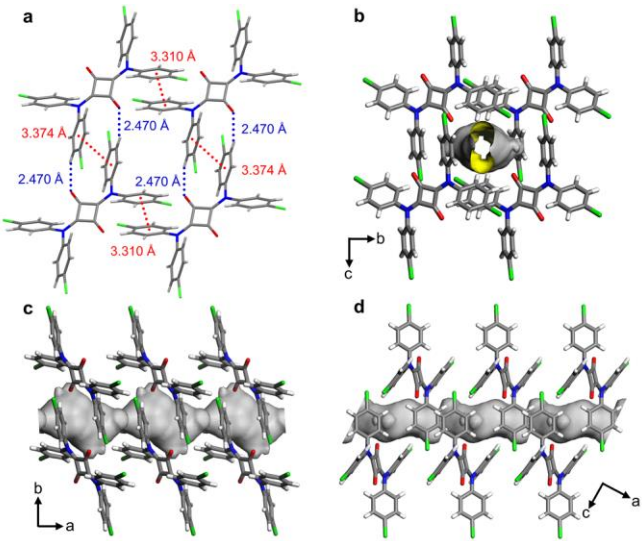


**Figure S6.** (a) The intermolecular interactions between SPOC-SQ molecules for the framework of SPOC-SQ-$C_2H_4$. (b-d) Packing diagrams of SPOC-SQ-$C_2H_4$ with the solvent-accessible void space visualized by yellow/grey (inner/outer) curved planes generated with a probe of 1.4 Å. Gray, blue, red, green, and white represent C, N, O, Cl, and H, respectively.

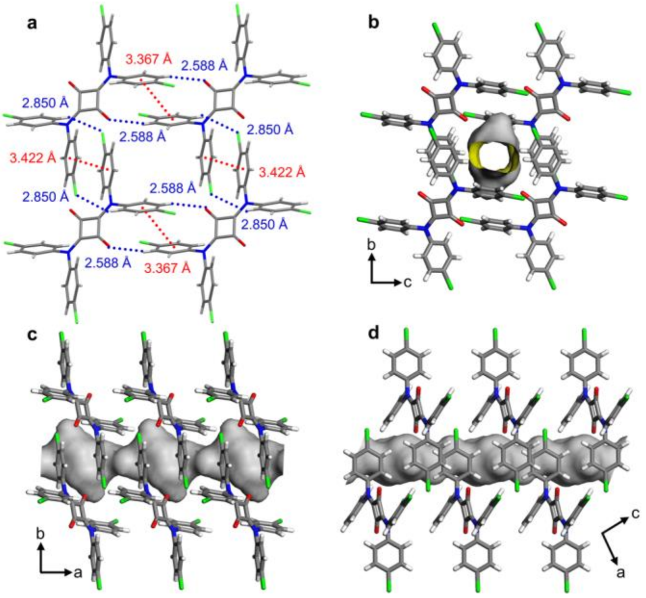


**Figure S7.** (a) The intermolecular interactions between SPOC-SQ molecules for the framework of SPOC-SQ-$C_2H_6$. (b-d) packing diagrams of SPOC-SQ-$C_2H_6$ with the solvent-accessible void space visualized by yellow/grey (inner/outer) curved planes generated with a probe of 1.4 Å. Gray, blue, red, green, and white represent C, N, O, Cl, and H, respectively.

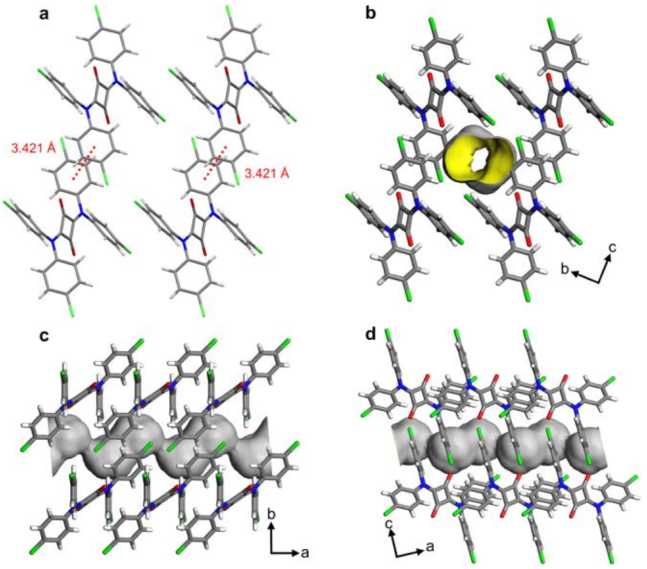


**Figure S8.** (a) The intermolecular interactions between SPOC-SQ molecules for the framework of SPOC-SQ-$C_3H_4$. (b-d) Packing diagrams of SPOC-SQ-$C_3H_4$ with the solvent-accessible void space visualized by yellow/grey (inner/outer) curved planes generated with a probe of 1.4 Å. Gray, blue, red, green, and white represent C, N, O, Cl, and H, respectively.

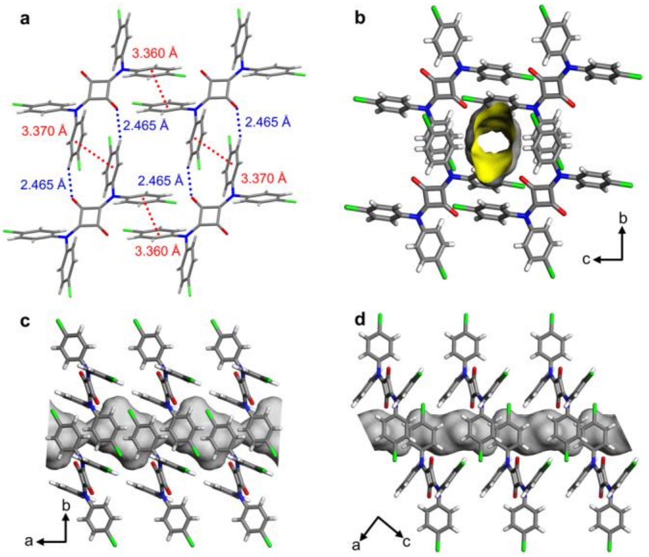


**Figure S9.** (a) The intermolecular interactions between SPOC-SQ molecules for the framework of SPOC-SQ-$C_3H_6$. (b-d) Packing diagrams of SPOC-SQ-$C_3H_6$ with the solvent-accessible void space visualized by yellow/grey (inner/outer) curved planes generated with a probe of 1.4 Å. Gray, blue, red, green, and white represent C, N, O, Cl, and H, respectively.

**Table S8**. The molecular sizes and kinetic diameters for $CO_2$, $C_2H_2$, $C_2H_4$, $C_2H_6$, $C_3H_4$, and $C_3H_6$.

| | | Molecular size (Å$^3$) | Kinetic diameter (Å) |
|---|---|---|---|
| $CO_2$ | 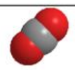 | 3.18 × 3.33 × 5.36 | 3.3 |
| $C_2H_2$ | 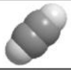 | 3.32 × 3.34 × 5.70 | 3.3 |
| $C_2H_4$ | 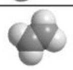 | 3.28 × 4.18 × 4.84 | 4.2 |
| $C_2H_6$ | 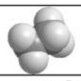 | 3.81 × 4.08 × 4.82 | 4.4 |
| $C_3H_4$ | 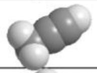 | 4.01 × 4.16 × 6.51 | 4.2 |
| $C_3H_6$ | 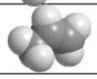 | 4.16 × 4.65 × 6.44 | 4.7 |

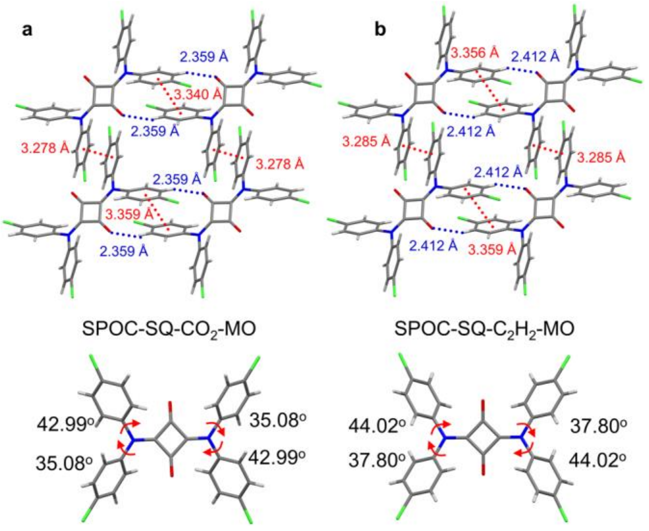


**Figure S10**. The intermolecular interactions between SPOC-SQ molecules and molecular conformations for the gate-opened frameworks of (a) SPOC-SQ-$CO_2$-MO and (b) SPOC-SQ-$C_2H_2$-MO obtained after removal of linear gases. Gray, blue, red, green, and white represent C, N, O, Cl, and H, respectively.

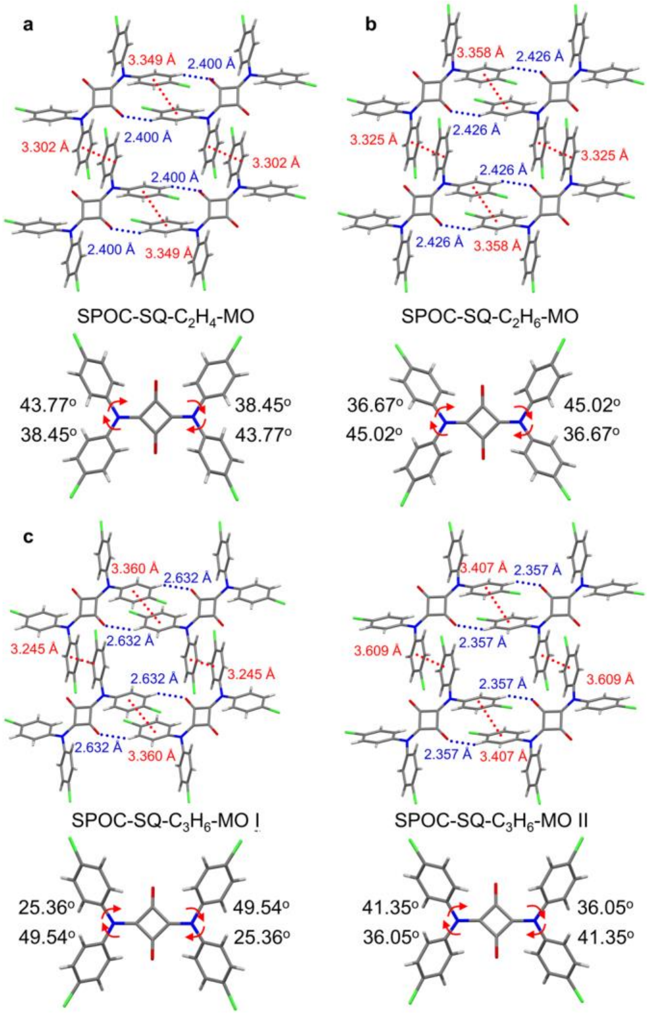

**Figure S11**. The intermolecular interactions between SPOC-SQ molecules and molecular conformations for the gate-opened frameworks of (a) SPOC-SQ-$C_2H_4$-MO, (b) SPOC-SQ-$C_2H_6$-MO, and (c) SPOC-SQ-$C_3H_6$-MO obtained after removal of nonlinear gases. Among them, there are two kinds of molecular conformations in SPOC-SQ-$C_3H_6$-MO (I and II) due to the largest disturbance of $C_3H_6$ to the framework, the average distances of π-π interactions in these two situations were used to analyze. Gray, blue, red, green, and white represent C, N, O, Cl, and H, respectively.

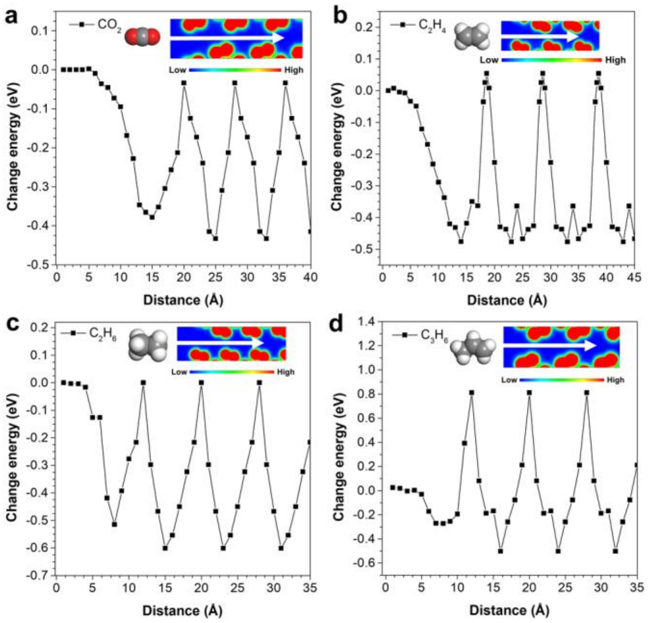


**Figure S12.** Potential energy profiles of (a) $CO_2$, (b) $C_2H_4$, (c) $C_2H_6$, and (d) $C_3H_6$ entering SPOC-SQ-gas framework along the central axis of the 1D channel. Insets show the profile of the 1D channel based on the total electron density derived from the DFT calculation. Gray, red, and white represent C, O, and H, respectively.

**Table S9.** Summary of the maximum and minimum energies and the difference between them upon gas molecules entering 1D channels.

| Gas | Max. energy (eV) | Min. energy (eV) | Energy difference value (eV) |
|---|---|---|---|
| $CO_2$ | -0.03418 | -0.43245 | 0.39827 |
| $C_2H_4$ | 0.05437 | -0.47719 | 0.53156 |
| $C_2H_6$ | -0.00009 | -0.60146 | 0.60137 |
| $C_3H_6$ | 0.81178 | -0.50275 | 1.31453 |

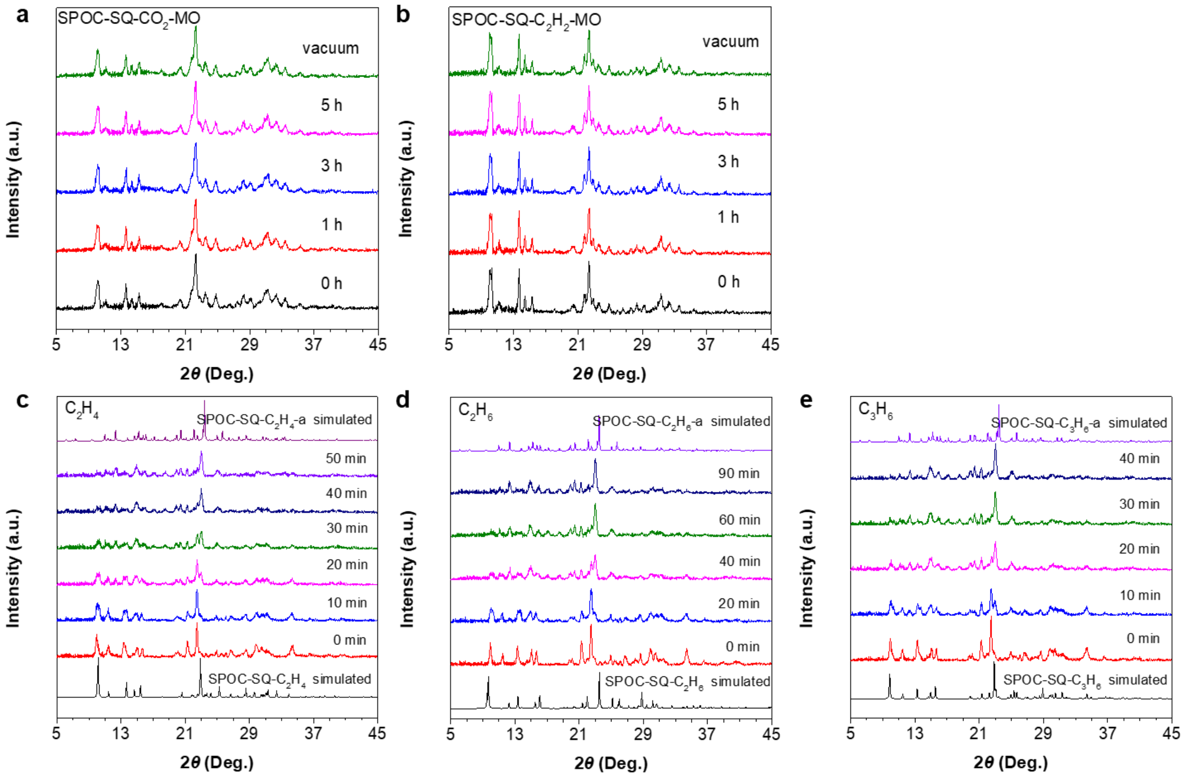


**Figure S13.** *In-situ* PXRD patterns of (a) SPOC-SQ-$CO_2$-MO and (b) SPOC-SQ-$C_2H_2$-MO at room temperature under air or vacuum for different time. *In-situ* PXRD patterns of (c) SPOC-SQ-$C_2H_4$, (d) SPOC-SQ-$C_2H_6$, and (e) SPOC-SQ-$C_3H_6$ single crystals after exposure to room temperature in air for different times, and their simulated PXRD patterns.

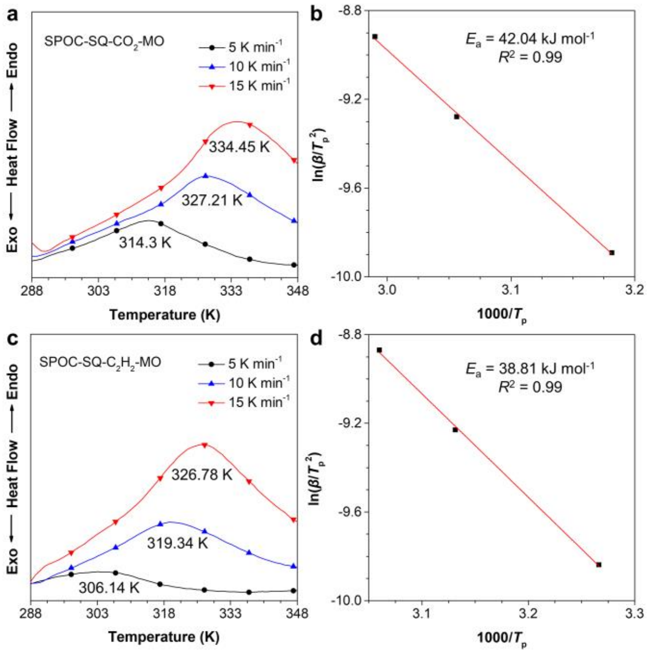

**Figure S14.** (a and c) DSC traces of (a) SPOC-SQ-$CO_2$-MO and (c) SPOC-SQ-$C_2H_2$-MO in the first heating process at heating rates of 5, 10, and 15 K min$^{-1}$. (b and d) Kissinger plots for the phase transitions of (b) SPOC-SQ-$CO_2$-MO and (d) SPOC-SQ-$C_2H_2$-MO. In the Kissinger method, $\beta$ and $T_p$ denote heating rate and temperature at the maximum of the DSC peak due to the transformation, respectively, and $E_a$ denotes activation energy.

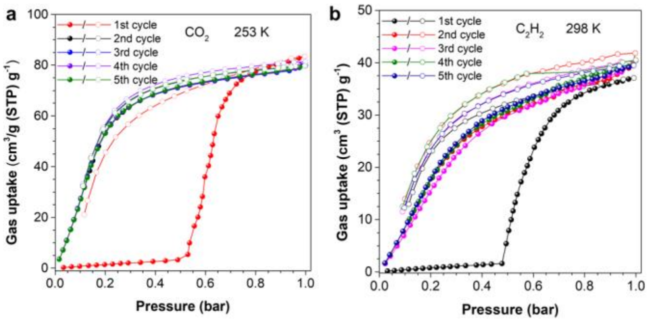


**Figure S15**. Sorption isotherms of SPOC-SQ-a microcrystals for (a) $CO_2$ at 253 K and (b) $C_2H_2$ at 298 K for the first to fifth adsorption-desorption processes. The sample was vacuumed before the second adsorption-desorption processes. Filled and open circle symbols represent adsorption and desorption, respectively.

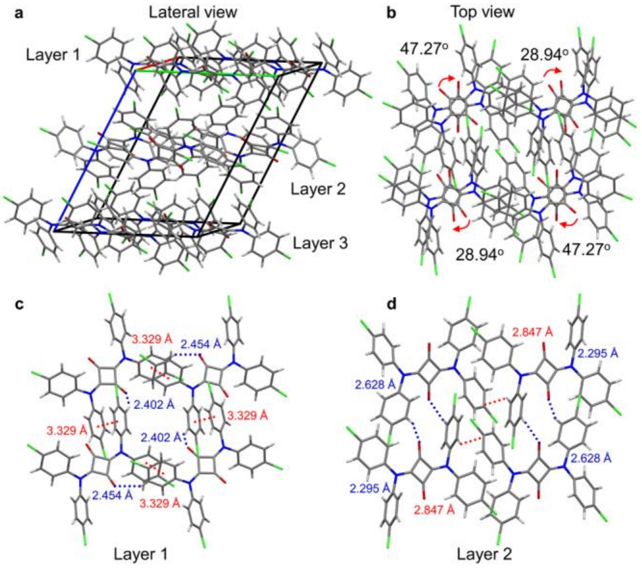


**Figure S16**. (a) Lateral and (b) top views of the packing diagrams for SPOC-SQ-a after $CO_2$ removal. (c and d) Intermolecular interactions between SPOC-SQ molecules in (c) layer 1 and (d) layer 2 for SPOC-SQ-a after $CO_2$ removal. Gray, blue, red, green, and white represent C, N, O, Cl, and H, respectively.

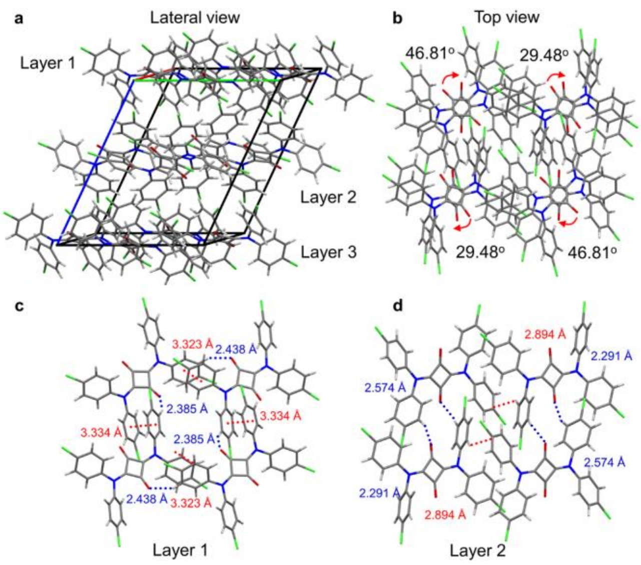


**Figure S17**. (a) Lateral and (b) top views of the packing diagrams for SPOC-SQ-a after $C_2H_2$ removal. (c and d) Intermolecular interactions between SPOC-SQ molecules in (c) layer 1 and (d) layer 2 for SPOC-SQ-a after $C_2H_2$ removal. Gray, blue, red, green, and white represent C, N, O, Cl, and H, respectively.

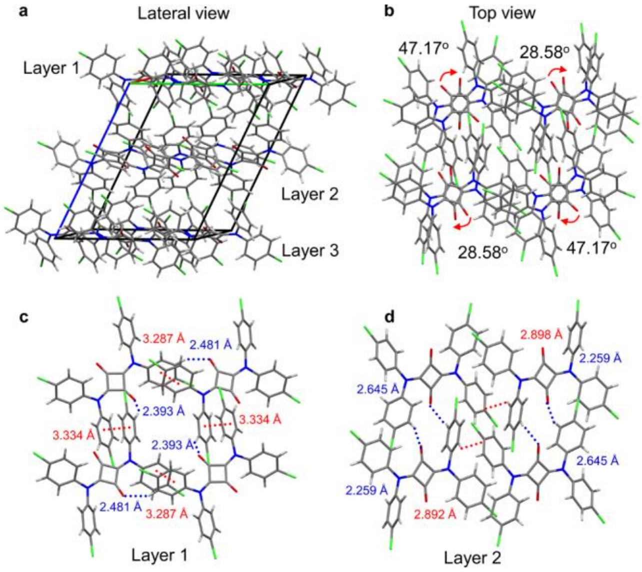


**Figure S18**. (a) Lateral and (b) top views of the packing diagrams for SPOC-SQ-a after $C_2H_4$ removal. (c and d) Intermolecular interactions between SPOC-SQ molecules in (c) layer 1 and (d) layer 2 for SPOC-SQ-a after $C_2H_4$ removal. Gray, blue, red, green, and white represent C, N, O, Cl, and H, respectively.

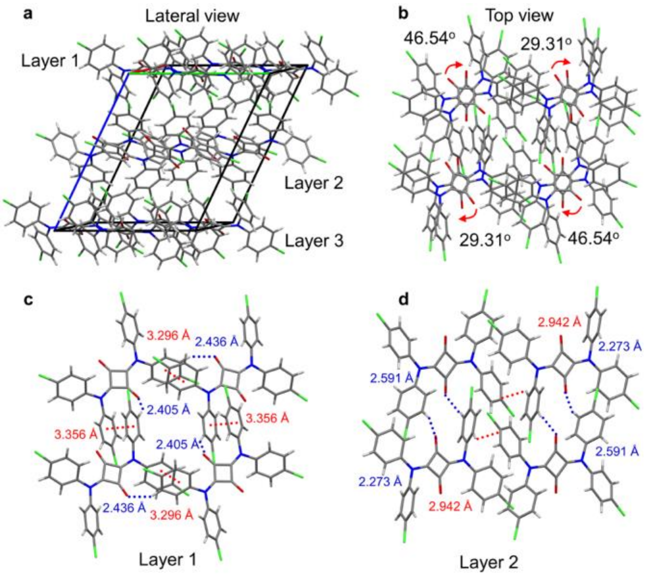


**Figure S19**. (a) Lateral and (b) top views of the packing diagrams for SPOC-SQ-a after $C_2H_6$ removal. (c and d) Intermolecular interactions between SPOC-SQ molecules in (c) layer 1 and (d) layer 2 for SPOC-SQ-a after $C_2H_6$ removal. Gray, blue, red, green, and white represent C, N, O, Cl, and H, respectively.

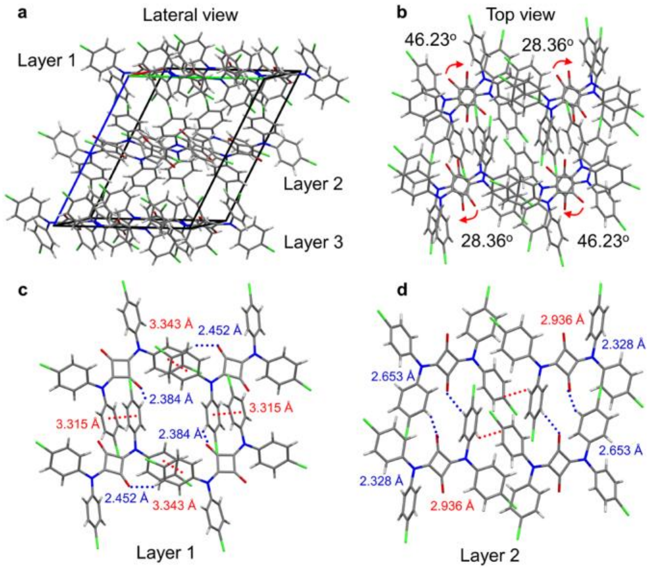


**Figure S20**. (a) Lateral and (b) top views of the packing diagrams for SPOC-SQ-a after $C_3H_6$ removal. (c and d) Intermolecular interactions between SPOC-SQ molecules in (c) layer 1 and (d) layer 2 for SPOC-SQ-a after $C_3H_6$ removal. Gray, blue, red, green, and white represent C, N, O, Cl, and H, respectively.

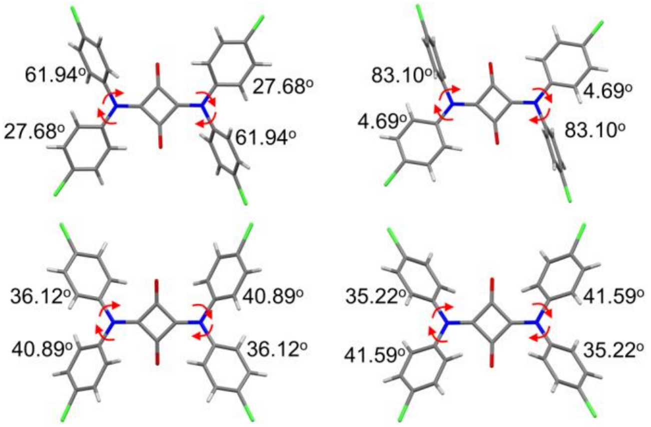


**Figure S21**. Molecular conformations of SPOC-SQ molecules in SPOC-SQ-a after $CO_2$ removal. Gray, blue, red, green, and white represent C, N, O, Cl, and H, respectively.

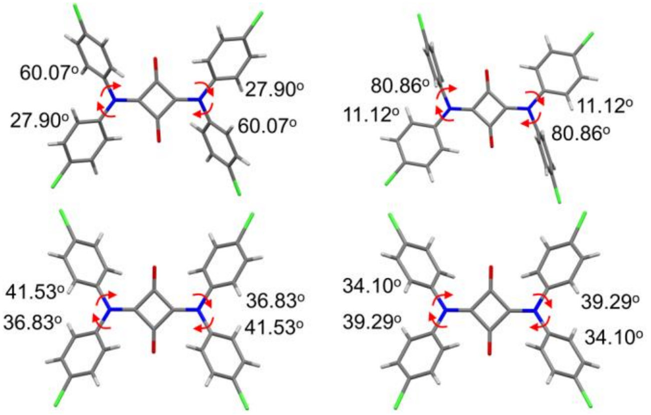


**Figure S22**. Molecular conformations of SPOC-SQ molecules in SPOC-SQ-a after $C_2H_2$ removal. Gray, blue, red, green, and white represent C, N, O, Cl, and H, respectively.

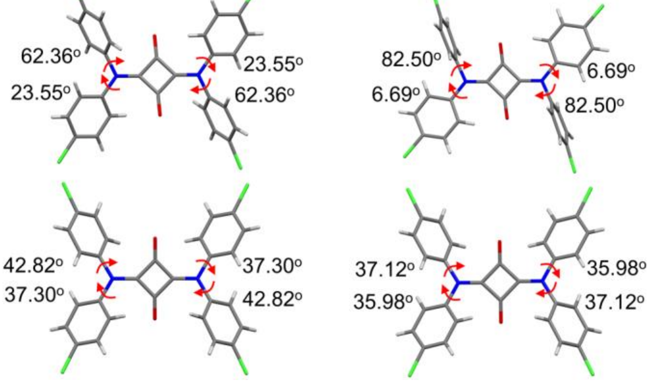


**Figure S23**. Molecular conformations of SPOC-SQ molecules in SPOC-SQ-a after $C_2H_4$ removal. Gray, blue, red, green, and white represent C, N, O, Cl, and H, respectively.

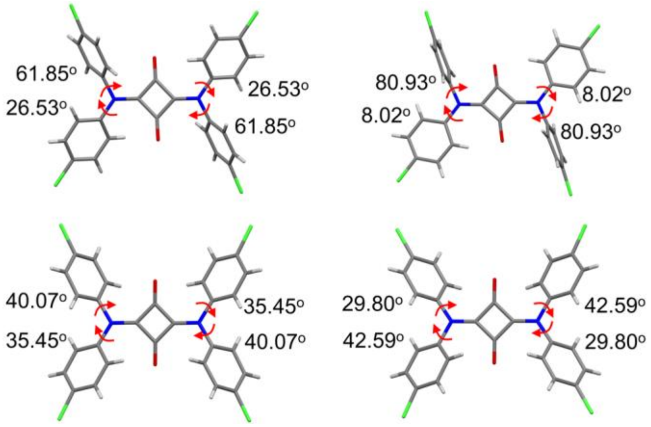


**Figure S24**. Molecular conformations of SPOC-SQ molecules in SPOC-SQ-a after $C_2H_6$ removal. Gray, blue, red, green, and white represent C, N, O, Cl, and H, respectively.

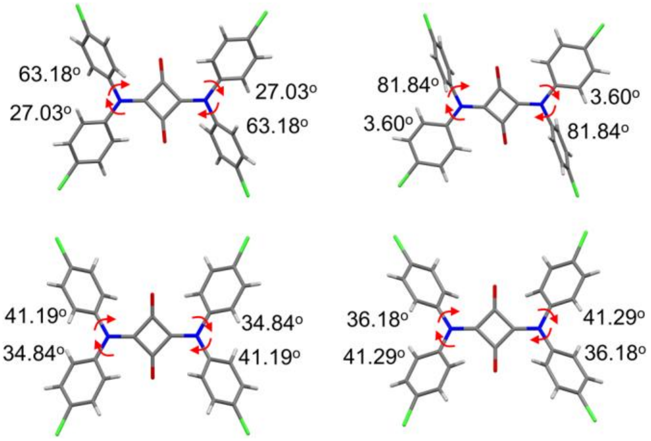


**Figure S25**. Molecular conformations of SPOC-SQ molecules in SPOC-SQ-a after $C_3H_6$ removal. Gray, blue, red, green, and white represent C, N, O, Cl, and H, respectively.

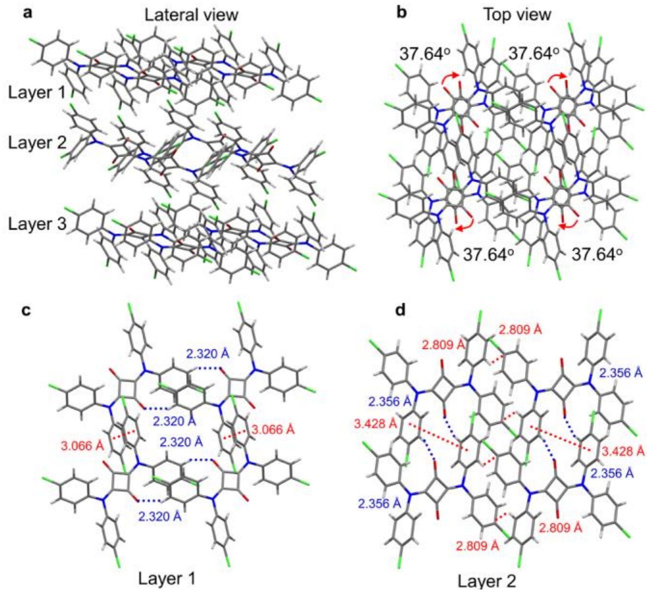


**Figure S26**. (a) Lateral and (b) top views of the packing diagrams for SPOC-SQ-$C_2H_4$-MSO after $C_2H_4$ removal. (c and d) Intermolecular interactions between SPOC-SQ molecules in (c)

layer 1 and (d) layer 2 for SPOC-SQ-$C_2H_4$-MSO after $C_2H_4$ removal. Gray, blue, red, green, and white represent C, N, O, Cl, and H, respectively.

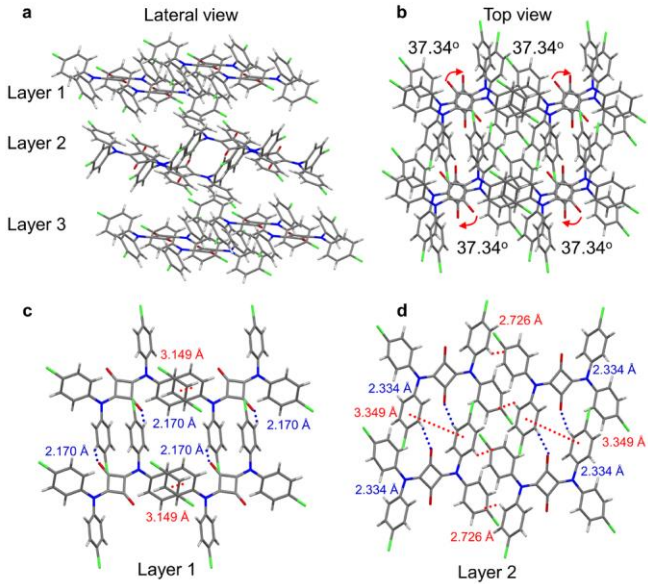


**Figure S27**. (a) Lateral and (b) top views of the packing diagrams for SPOC-SQ-$C_2H_6$-MSO after $C_2H_6$ removal. (c and d) Intermolecular interactions between SPOC-SQ molecules in (c) layer 1 and (d) layer 2 for SPOC-SQ-$C_2H_6$-MSO after $C_2H_6$ removal. Gray, blue, red, green, and white represent C, N, O, Cl, and H, respectively.

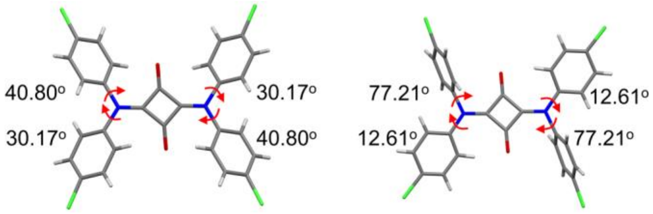


**Figure S28**. Molecular conformations of SPOC-SQ molecules in SPOC-SQ-$C_2H_4$-MSO after $C_2H_4$ removal. Gray, blue, red, green, and white represent C, N, O, Cl, and H, respectively.

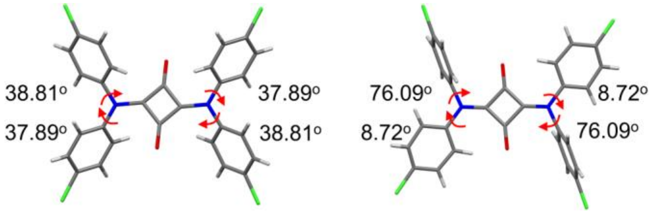


**Figure S29**. Molecular conformations of SPOC-SQ molecules in SPOC-SQ-$C_2H_6$-MSO after $C_2H_6$ removal. Gray, blue, red, green, and white represent C, N, O, Cl, and H, respectively.

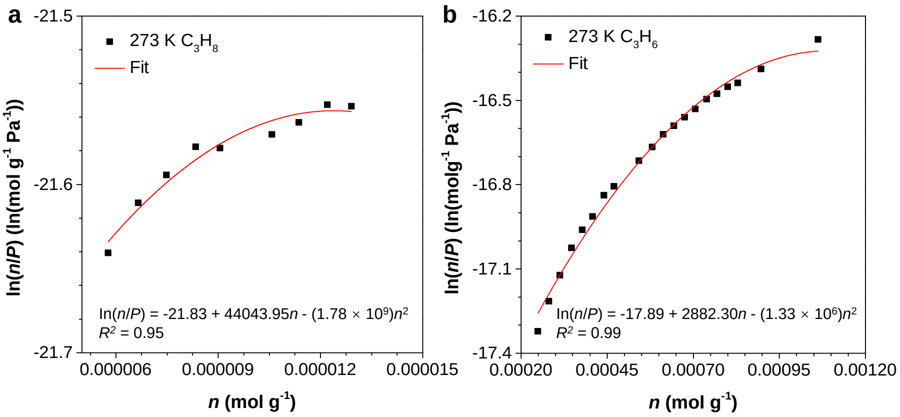


**Figure S30.** The virial graphs for adsorption of (a) $C_3H_8$ and (b) $C_3H_6$ on SPOC-SQ-a crystals at 273 K.

**Table S10**. Summary of virial parameters ($A_0$: adsorbate-adsorbent interactions; $A_1$: adsorbate-adsorbate interactions; $R^2$: variance; $K_H$: Henry's law constant), selectivity of gas sorption ($S_{i/C3H8}$: selectivity of $C_3H_6$ over $C_3H_8$) on SPOC-SQ-a at 273 K obtained from the virial equation.

| Gas | *T* (K) | $A_0$ (ln(molg$^{-1}$Pa$^{-1}$)) | $A_1$ (g mol$^{-1}$) | $A_2$ (g mol$^{-1}$) | $R^2$ | $K_H$ | $S_{i/C3H8}$ |
|---|---|---|---|---|---|---|---|
| $C_3H_6$ | 273 | -17.89 ± 0.044 | 2883.30 ± 151.30 | $1.32\times10^6$ ± 121028.12 | 0.99 | $1.69\times10^{-8}$ | 51.16 |
| $C_3H_8$ | 273 | -21.83 ± 0.04 | 44043.95 ± 9082.95 | $1.78\times10^9$ ± $4.81\times10^8$ | 0.95 | $3.31\times10^{-10}$ | 1 |

## References

[1] J. VandeVondele, M. Krack, F. Mohamed, M. Parrinello, T. Chassaing, J. Hutter, *Comput. Phys. Commun.* **2005**, *167*, 103-128.

[2] S. Goedecker, M. Teter, J. Hutter, *Phys. Rev. B* **1996**, *54*, 1703-1710.

[3] M. Krack, *Chem. Acc.* **2005**, *114*, 145-152.

[4] J. VandeVondele, J. Hutter, *J. Chem. Phys.* **2007**, *127*, 114105.

[5] J. SMoellmann, S. Grimme, *J. Phys. Chem. C* **2014**, *118*, 7615-7621.
